# Structural basis of light‐harvesting in the photosystem II core complex

**DOI:** 10.1002/pro.3841

**Published:** 2020-02-24

**Authors:** Frank Müh, Athina Zouni

**Affiliations:** ^1^ Department of Theoretical Biophysics, Institute for Theoretical Physics Johannes Kepler University Linz Linz Austria; ^2^ Humboldt‐Universität zu Berlin, Institute for Biology, Biophysics of Photosynthesis Berlin Germany

**Keywords:** carotenoid, charge separation, chlorophyll, cryo‐electron microscopy, excitation energy transfer, reaction center, X‐ray crystallography

## Abstract

Photosystem II (PSII) is a membrane‐spanning, multi‐subunit pigment–protein complex responsible for the oxidation of water and the reduction of plastoquinone in oxygenic photosynthesis. In the present review, the recent explosive increase in available structural information about the PSII core complex based on X‐ray crystallography and cryo‐electron microscopy is described at a level of detail that is suitable for a future structure‐based analysis of light‐harvesting processes. This description includes a proposal for a consistent numbering scheme of protein‐bound pigment cofactors across species. The structural survey is complemented by an overview of the state of affairs in structure‐based modeling of excitation energy transfer in the PSII core complex with emphasis on electrostatic computations, optical properties of the reaction center, the assignment of long‐wavelength chlorophylls, and energy trapping mechanisms.

## 1 INTRODUCTION

Many organisms use solar energy in a very efficient way, and it is of great interest to understand, how they can achieve this.^1^, ^2^, ^3^, ^4^ Cyanobacteria, algae, and plants carry out oxygenic photosynthesis.^5^, ^6^, ^7^ In this variant, two light‐powered molecular machines^8^, ^9^ known as photosystem I (PSI)^10^, ^11^ and photosystem II (PSII)^12^, ^13^, ^14^, ^15^, ^16^, ^17^, ^18^, ^19^ operate in series to ultimately transfer electrons from water to nicotinamide adenine dinucleotide phosphate (NADPH in its reduced form) and to produce adenosine triphosphate. Both these molecules are required to transform carbon dioxide (CO_2_) into biomass.^1^, ^6^, ^20^, ^21^

**Figure 1:**
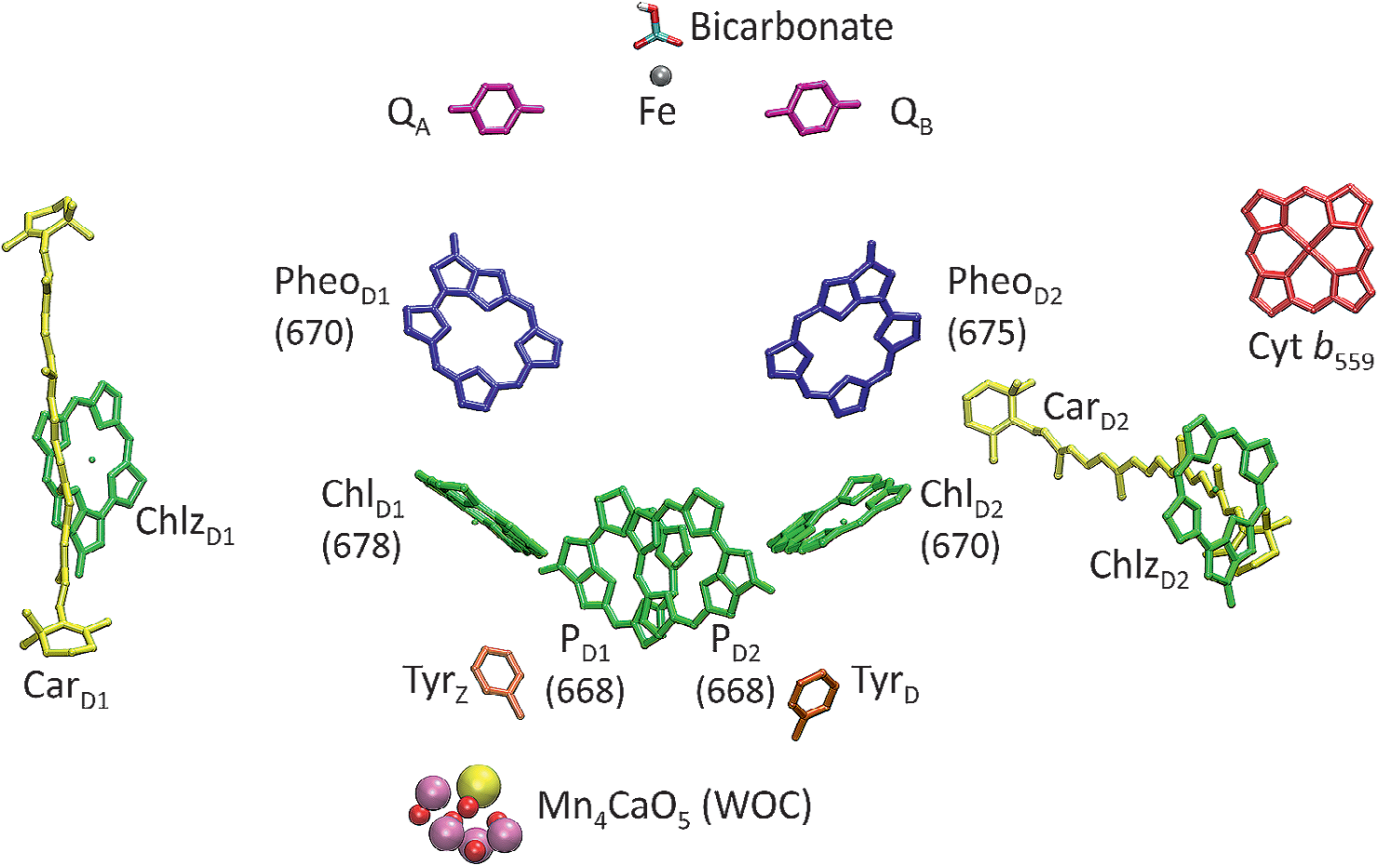
Arrangement of cofactors in and around the RC of PSII (Chl *a*: P_D1_, P_D2_, Chl_D1_, Chl_D2_, Chlz_D1_, Chlz_D2_; green. Pheo *a*: Pheo_D1_, Pheo_D2_, blue. Plastoquinone: Q_A_, Q_B_, magenta. β‐Carotene: Car_D1_, Car_D2_; yellow. Heme: cytochrome *b*_559_ 29; red. Nonheme iron: Fe with bound bicarbonate.^30^, ^31^ WOC: Mn_4_CaO_5_‐cluster. Redox‐active tyrosines^14^: Tyr_Z_, Tyr_D_; orange). Substituents are largely omitted for clarity. The numbers in parentheses refer to the site energies (in nm) assigned to the RC pigments on the basis of electrostatic computations.^28^ Figure made with VMD^32^ based on PDB ID 6DHE^26^ (cf. Table 1)

The oxidation of water actually takes place in PSII, which initially transfers the electrons to plastoquinone (PQ) and thus is a water:PQ oxidoreductase. PSII is a multi‐subunit membrane protein complex situated in the thylakoid membrane,^8^, ^22^, ^23^ and it releases the reduced PQ (plastoquinol, PQH_2_) into that membrane,^17^ where it diffuses to another membrane protein complex (cytochrome *b*_6_ *f*) to deliver the electrons.^24^ The oxidation reaction of water is catalyzed by a protein‐bound manganese (i.e., Mn_4_CaO_5_) complex known as the water‐oxidizing complex (WOC; or oxygen‐evolving complex). In recent years, much effort has been put toward unraveling the structure and inner workings of the WOC,^7^, ^15^, ^25^ and the struggle still goes on.^26^, ^27^ The WOC is situated next to the reaction center (RC) of PSII. A remarkable feature of the RC is that it is structurally symmetric but functionally asymmetric.^28^ There is a pseudo‐C_2_‐symmetric array of redox‐active cofactors, including chlorophylls (Chls), pheophytins (Pheos), and two PQs (see below for further details, Figure 1 for a first glance at the structure, and our earlier review^13^ for chemical structures). They are held in place by the heterodimeric structure of the two protein subunits PsbA (also known as D1 protein) and PsbD (D2 protein). This protein scaffold modifies the properties of the cofactors in a way that they are able to transform the electronically excited states of the tetrapyrroles (i.e., the Chls and the Pheos) into charge‐separated states, whereby only one of the two branches is used for electron transfer (ET), and, finally, the WOC gets oxidized and one of the PQs reduced.^14^, ^16^, ^17^, ^30^, ^31^

The Chls and Pheos in the RC (Figure 1) are not sufficient for an efficient transformation of solar energy under natural conditions.^1^ Therefore, each RC is associated with additional pigment–protein complexes (PPCs) whose task is the extensive absorption of photons and the delivery of the energy to the RC by virtue of excitation energy transfer (EET).^49^, ^50^, ^51^, ^52^ These PPCs are referred to as antenna proteins or light‐harvesting complexes. Basically, one can differentiate between core antennae, being tightly bound to the RC and forming a stoichiometrically fixed unit with the RC that is termed the *core complex* (cc), and peripheral antennae, whose amount can vary with light conditions thus defining a means of regulating EET.^1^ In the present review, we focus on the photosystem II core complex (PSIIcc) containing the RC and the two core antennae known as CP43 and CP47. However, understanding light harvesting in PSIIcc also requires considering the supercomplex arrangement in the thylakoid membrane forming the environment of the core complex, which we will briefly describe based on the presently available structures (Table 1).

**Table 1:** Structures of the photosystem II core complex (cc) with a resolution ≤3.02 Å available in the RCSB Protein Data Bank as of August 2019

PDB ID	Complex type	Organism type	Organism	Res. (Å)	Method^a^	Subunit composition of PSIIcc^b^	References
2AXT	cc^c^	Cyanob.	*Th. elongatus* d	3.0	XRD	M, O, T, U, V, Z, X_1_ e, X_2_ e, X_3_ e	33
4V62^f^	cc^c^	Cyanob.	*Th. elongatus* d	2.9	XRD	M, O, T, U, V, X, Y^e^, Z, 30	34
3WU2^g^	cc^c^	Cyanob.	*Th. vulcanus*	1.9	XRD	M, O, T, U, V, X, Z, 30	35
4IL6	cc, Sr^h^	Cyanob.	*Th. vulcanus*	2.1	XRD	M, O, T, U, V, X, Y^i^, Z, 30	36
4PJ0	cc^c^^,^ j	Cyanob.	*Th. elongatus* d	2.44	XRD	M, O, T, U, V, X, Y, Z, 30	37
4UB6	cc^c^	Cyanob.	*Th. vulcanus*	1.95	XRD, fs	M, O, T, U, V, X, Y^i^, Z, 30	38
4UB8	cc^c^	Cyanob.	*Th. vulcanus*	1.95	XRD, fs	M, O, T, U, V, X, Y^i^, Z, 30	38
4YUU	cc^c^^,^ k	Red alga	*C. caldarium*	2.76	XRD	M, O, Q´^l^, T, U, V, W^e^, X, Z^l^, 30, 34^e^	39
5KAF	cc^c^^,^ j	Cyanob.	*Th. elongatus* d	3.0	RT SFX	M, O, T, U, V, X, Y, Z, 30	40
5KAI	cc^j^, NH_3_ m	Cyanob.	*Th. elongatus* d	2.8	RT SFX	M, O, T, U, V, X, Y, Z, 30	40
5TIS	cc^j^, 2F^n^	Cyanob.	*Th. elongatus* d	2.25	RT SFX	M, O, T, U, V, X, Y, Z, 30	40
5B5E	cc^c^	Cyanob.	*Th. vulcanus*	1.87	XRD	M, O, T, U, V, X, Z, 30	41
5B66	cc^c^	Cyanob.	*Th. vulcanus*	1.85	XRD	M, O, T, U, V, X, Z, 30	41
5GTH	cc^c^	Cyanob.	*Th. vulcanus*	2.5	RT SFX	M, O, T, U, V, X, Y^i^, Z, 30	42
5GTI	cc, 2F^n^	Cyanob.	*Th. vulcanus*	2.5	RT SFX	M, O, T, U, V, X, Y^i^, Z, 30	42
5WS5	cc^o^	Cyanob.	*Th. vulcanus*	2.35	RT SFX	M, O, T, U, V, X, Y^i^, Z, 30	42
5WS6	cc^o^, 2F^n^	Cyanob.	*Th. vulcanus*	2.35	RT SFX	M, O, T, U, V, X, Y^i^, Z, 30	42
5H2F	cc, ΔM^p^	Cyanob.	*Th. elongatus* d	2.2	XRD	O, T, U, V, X, Z, 30	43
5MX2	cc, apo^q^	Cyanob.	*Th. elongatus* d	2.55	XRD	M, O, T, U, V, X, Y, Z, 30	44
5V2C^r^	cc^c^	Cyanob.	*Th. vulcanus*	1.9	XRD	M, O, T, U, V, X, Z, 30	45
5XNL	C_2_S_2_M_2_ s	Plant	*Pisum sativum*	2.7	EM	M, O, P, Q, T, W, X, Z	46
5ZZN	cc, ΔsqdB^t^	Cyanob.	*Th. elongatus* d	2.1	XRD	M, O, T, U, V, X, Z, 30	47
6DHE	cc^c^^,^ j	Cyanob.	*Th. elongatus* d	2.05	RT SFX	M, O, T, U, V, X, Y, Z, 30	26
6DHF	cc^j^, 1F^u^	Cyanob.	*Th. elongatus* d	2.08	RT SFX	M, O, T, U, V, X, Y, Z, 30	26
6DHG	cc^j^, 2F^v^	Cyanob.	*Th. elongatus* d	2.5	RT SFX	M, O, T, U, V, X, Y, Z, 30	26
6DHH	cc^j^, 2F^w^	Cyanob.	*Th. elongatus* d	2.2	RT SFX	M, O, T, U, V, X, Y, Z, 30	26
6DHO	cc^j^, 2F^x^	Cyanob.	*Th. elongatus* d	2.07	RT SFX	M, O, T, U, V, X, Y, Z, 30	26
6DHP	cc^j^, 3F^y^	Cyanob.	*Th. elongatus* d	2.04	RT SFX	M, O, T, U, V, X, Y, Z, 30	26
6JLU	PSII‐FCP^z^	Diatom	*Ch. gracilis*	3.02	EM	G, M, O, Q´, T, U, V, W, X, Z, 30, 31, 34	48

a XRD: standard X‐ray crystallography with synchrotron source; XRD, fs: X‐ray crystallography with XFEL source; RT SFX: room temperature serial fs X‐ray crystallography with XFEL source; EM: cryo‐electron microscopy.

b The subunits PsbA (D1‐protein), PsbB (CP47), PsbC (CP43), PsbD (D2‐protein), PsbE (α‐subunit of cyt b_559_), PsbF (β‐subunit of cyt b_559_), PsbH, PsbI, PsbJ, PsbK, and PsbL are present in all structures and not listed. For listed subunits, the prefix “Psb” is omitted.

c Dark‐adapted PSIIcc without modifications.

d Strain BP‐1.

e Unassigned or incompletely modeled subunit.

f Supersedes 3BZ1, 3BZ2.

g Supersedes 3ARC.

h Ca^2+^ in the WOC replaced by Sr^2+^ biosynthetically.

i Present in one monomer.

j New crystal form of cyanobacterial PSIIcc with dimers forming native‐like rows in the crystal.

k PSIIcc forms tetramers in the crystal.

l Present in two monomers.

m Ammonia bound to the WOC and two‐flash illuminated (S_3_‐enriched).

n Two‐flash illuminated (S_3_‐enriched).

o Pre‐flashed (see Suga et al.^42^).

p PsbM deletion mutant.

q Depleted of the Mn_4_CaO_5_‐cluster after crystallization.

r Refinement of 3ARC/3WU2.

s Dimeric supercomplex containing PSIIcc, LHCII, CP24, CP26, and CP29.

t SQDG deletion mutant.

u One‐flash illuminated (S_2_‐rich).

v Two‐flash illuminated, after 150 μs.

w Two‐flash illuminated, after 400 μs.

x Two‐flash illuminated (S_3_‐rich).

y Three‐flash illuminated (S_0_‐rich).

z Dimeric supercomplex containing PSIIcc and fucoxanthin‐Chl‐a/c‐binding proteins (FCPs).

While knowing the spatial structure of an antenna system is a prerequisite for uncovering its working principles, the structure can be linked to function only by theoretical modeling. As in our earlier review,^13^ the description of the structural basis will be followed by an overview of structure‐based computations, where the focus is on the electrostatic methods developed in the Linz group.^28^, ^51^, ^53^ Readers interested in other methods elaborating on quantum chemistry (QC) are referred to recent reviews by Mennucci and coworkers.^54^, ^55^

## 2 OVERALL STRUCTURE OF PSII

Our earlier review about the antenna structure of PSIIcc^13^ is based on the crystal structure by Loll et al.^33^ at a resolution of 3.0 Å. Since then, the number of structures with a better resolution has literally exploded (Table 1). This suitable development is on one hand due to the improvement of biochemical preparations and crystallization methods and on the other hand due to technological innovations. The first small step toward an improved resolution was enabled by advances in computer software allowing for a reprocessing of the previous data at 3.0 to 2.9 Å resolution. The gain in insight was, however, significant, in particular as regards quinone diffusion channels, the role of lipids as cofactors, and chloride in the vicinity of the WOC^34^ (see other earlier reviews^17^, ^56^, ^57^, ^58^ for details concerning these developments).

A major breakthrough in the structural biology of PSII was achieved by Shen and coworkers who were able to improve the resolution of the crystal structure of cyanobacterial PSIIcc to a remarkable 1.9 Å.^35^ The crystal form was still the same as in the earlier work, but a suitable choice of detergents and post‐crystallization treatments paved the way to a real atomic resolution with important implications for an understanding of the WOC.^59^ With these developments, also started the era of the structural biology of systematically modified PSIIcc. The first example is a structure, where the Ca^2+^ ion of the WOC is replaced with Sr^2+^ biosynthetically.^36^

However, the mechanism behind the improvement of membrane protein crystals remained obscure, which is mainly because the underlying physical chemistry is notoriously difficult.^60^, ^61^ These problems also accompanied the parallel development of a new crystal form of cyanobacterial PSIIcc in our lab, which was actually found by chance. In an attempt to avoid unhinging of structurally relevant lipids^57^, ^58^ from PSIIcc, the detergent used to detach the complexes from the membrane was changed. Unexpectedly, the post‐crystallization treatments of the new crystals aiming at a dehydration to improve packing and resolution not only led to an extraction of water, but also to a detergent depletion. As a result, the PSIIcc dimers in the crystal were repacked in a way that the resolution improved from 6.0 to 2.44 Å.^37^ Thus, a new route to high‐resolution structures of PSIIcc was found. The most recent results concerning the WOC are also based on this new crystal type.^26^, ^27^

**Figure 2:**
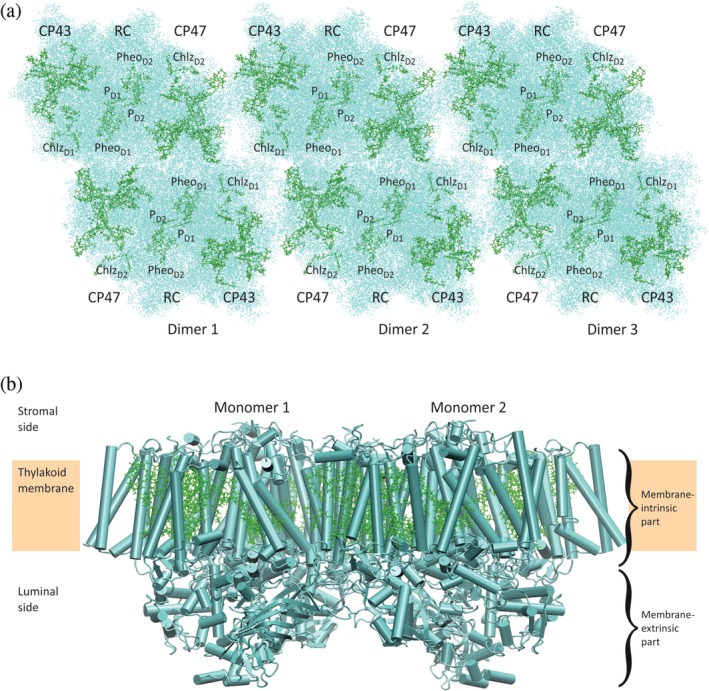
Structure of cyanobacterial PSIIcc. (a) View on a row of PSIIcc dimers (three dimers are shown) of *Thermosynechococcus elongatus* from the stromal side. The rows occur in a type of crystal first described by Hellmich et al.^37^ and are similar to PSIIcc superstructures found in native thylakoid membranes of cyanobacteria.^62^ Chls and Pheos are represented in CPK mode (green), the surrounding protein in points mode (cyan). (b) View on one PSIIcc dimer along the membrane plane illustrating the location of Chl pigments in the membrane and the significant membrane‐extrinsic extension of the protein (harboring the WOC close to the membrane) into the lumen. Chls and Pheos are represented in CPK mode (green), the surrounding protein backbone in cartoon mode (cyan). Figures made with VMD^32^ based on PDB ID 6DHE^26^ (cf. Table 1)

As regards light harvesting, the new crystal form of cyanobacterial PSIIcc has an appealing feature: The arrangement of dimers in a row (see Figure 2a) is similar to what is found in native thylakoid membranes of cyanobacteria.^37^ In fact, multiple rows often form extended two‐dimensional arrays of tightly packed dimers that are thought to be important in preventing spillover of excitation energy from PSII to PSI or direct energy flow from phycobilisomes to PSI.^62^ Phycobilisomes are the peripheral antennae of cyanobacteria.^1^ They contain open‐chain tetrapyrroles as pigments and are attached to both photosystems at the stromal side of the membrane.^63^, ^64^, ^65^ Besides their role in optimizing energy transfer from the phycobilisomes, the regular arrays may also facilitate energy exchange between core complexes in order to increase the efficiency of energy trapping. For example, an RC with reduced quinones cannot immediately use the excitation energy, that is, it is *closed*, and it takes a while until it is open again. In this time span, additional excitation energy arriving at the closed RC can flow back into the core antenna and be delivered to another, open RC in the array. While all this is hypothetical, the new crystal form provides a suitable structural basis for a simulation of these processes.

For completeness, we have to mention that there is a third crystal form of cyanobacterial PSIIcc. The two crystal forms discussed above have in common that the starting point is a solution of (detergent‐solubilized) *dimeric* core complexes (dPSIIcc), such as the one shown in Figure 2b, and that the crystals belong to space group P2_1_2_1_2_1_. They merely differ in the packing of dPSIIcc units and the detergent content, with the original crystal form being detergent‐rich and the new form detergent‐depleted. According to a general classification of membrane protein crystals,^66^ the former are of Type II and the latter of Type I. The third crystal form is also of Type II, but the starting point is a solution of *monomeric* core complexes (mPSIIcc), and the space group is C222_1_.^67^ Since the only structure obtained from this crystal form has a limited resolution of 3.6 Å, it is not listed in Table 1. Nonetheless, some features of this structure are of interest, as will be discussed below.

At the technological side, the last decade witnessed the development of the X‐ray free electron laser (XFEL) as a tool for structural biology.^27^, ^68^, ^69^ By using this X‐ray source, the first high‐resolution structure of dPSIIcc (1.95 Å) free of radiation damage due to reduction of the WOC was obtained.^38^ X‐ray induced radiation damage limits the structural information that can be extracted from a protein sample, and a dose limit of 2–3 × 10^7^ Gy (Gy = J kg^−1^) is recommended for cryo‐cooled protein crystals (77 K).^70^, ^71^ However, metal centers are prone to damage at much lower doses as they tend to capture photoelectrons, which has been directly demonstrated for the case of the WOC.^72^ An XFEL provides extremely short (<50 fs) and intense X‐ray pulses that, although destroying the sample completely, allow detecting a diffraction image before the onset of radiation damage. Thus, a large crystal can be scanned to obtain a “damage‐free” structure.

Yet, the actual advantage of the XFEL is that experiments can be performed at room temperature (RT) and with time resolution using a technique referred to as serial femtosecond X‐ray crystallography (SFX).^69^, ^73^ In this type of experiment, snapshot diffraction patterns are collected from randomly oriented microcrystals streamed across the X‐ray beam.^27^, ^74^ In the case of PSIIcc, the photochemistry in the RC leading to water oxidation and quinone reduction can be triggered by illuminating the microcrystals with light‐flashes. By carefully adjusting the number of flashes and the delay time between the last flash and the arrival of the crystal stream in the X‐ray beam, several states of the water oxidation and quinone reduction cycles can be probed. A number of data sets have been collected in this way,^26^, ^40^, ^42^ with the highest resolutions around 2 Å being obtained only recently.^26^ As detailed elsewhere,^27^ a prerequisite for these achievements was the production of a high amount of microcrystals with uniform cell constants. Since these microcrystals feature the native‐like superstructure (Figure 2a) and have been investigated at RT, we have now highly resolved structural information about cyanobacterial PSIIcc at our disposal that—in the framework of X‐ray crystallography—is as close as possible to physiological conditions. Indeed, it has been observed that the core complex is slightly expanded particularly along the membrane plane as compared with cryogenic structures.^26^, ^27^ In the future, these data will allow studying the effect of temperature‐dependent volume changes of the PPC on optical spectra and EET rates.

All X‐ray structures of PSIIcc are based on material from thermophilic cyanobacteria with one exception: Shen and coworkers succeeded in determining the structure of dPSIIcc from the (thermophilic and acidophilic) red alga *Cyanidium caldarium* at a resolution of 2.76 Å.^39^ Red algae are rather primitive eukaryotes in terms of evolution with a photosynthetic apparatus somewhere between cyanobacteria and chloroplasts.^1^, ^75^ Their PSIIcc is associated with phycobilisomes, whereas their PSIcc is surrounded by membrane‐intrinsic light‐harvesting complexes similar to plants. Despite the fact that the space group is the same as for the cyanobacterial dPSIIcc‐structures (P2_1_2_1_2_1_), the packing is different with PSIIcc forming tetramers in the crystal.^39^ However, these tetramers, in which two dPSIIcc units are stacked through their stromal surfaces, are unlikely to represent a native superstructure. The thylakoids of red algae are unstacked,^76^ and tetramers found are rather “double dimers,” in which two dPSIIcc units are attached to each other within the membrane like in the rows shown in Figure 2a. Double dimers are found in red algae^77^ and cyanobacteria.^62^ There are also indications for rows of dPSIIcc in the thylakoid membrane of red algae.^78^

**Figure 3:**
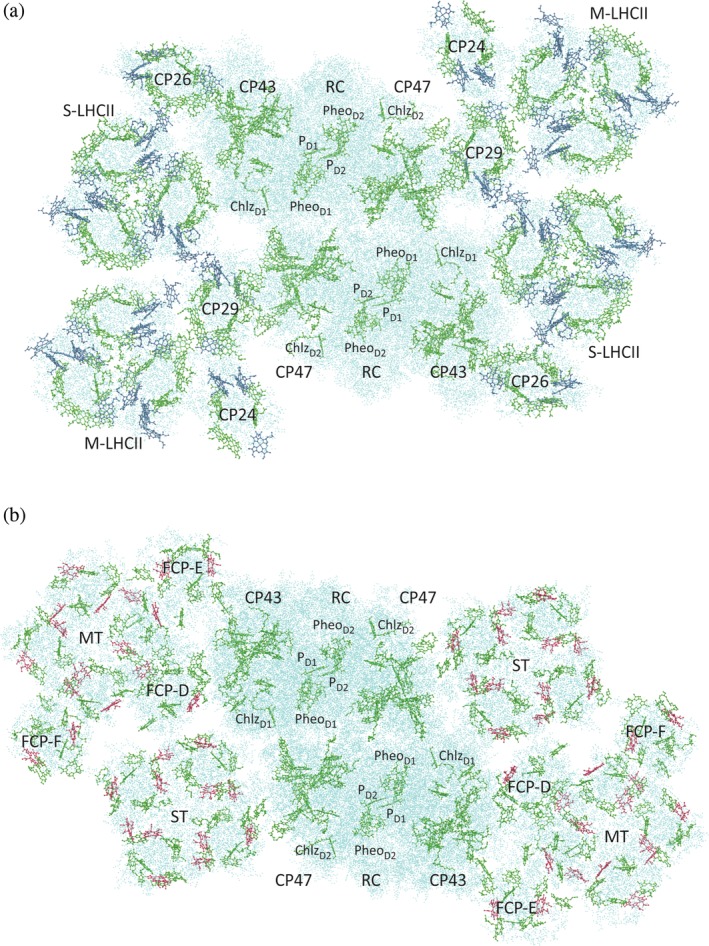
Structure of PSII supercomplexes from higher plants (a) and diatoms (b). (a) View on the C_2_S_2_M_2_ supercomplex of *Pisum sativum* from the stromal side. Chls and Pheos are represented in CPK mode (Chl *a* and Pheo *a* in green, Chl *b* in blue), the surrounding protein in points mode (cyan). Figure made with VMD^32^ based on PDB ID 5XNL^46^ (cf. Table 1). (b) View on the PSII‐FCPII supercomplex of *Chaetoceros gracilis* from the stromal side. Chls and Pheos are represented in CPK mode (Chl *a* and Pheo *a* in green, Chl *c* in red), the surrounding protein in points mode (cyan). Figure made with VMD^32^ based on PDB ID 6JLU^48^ (cf. Table 1)

The second remarkable technological advance in recent years is the “resolution revolution”^79^ in cryo‐electron microscopy (EM or cryo‐EM)^80^, ^81^, ^82^ permitting the structure elucidation of PSII supercomplexes from plants^46^ (Figure 3a) and, very recently, from diatoms^48^ (Figure 3b). In these supercomplexes, a dimer of core complexes is surrounded by peripheral antennae in a way that still leaves room for quinone exchange of the RC with the membrane phase. Structural and functional aspects of the peripheral antennae in plants have been described elsewhere.^18^, ^83^, ^84^, ^85^, ^86^, ^87^ Besides photoprotective regulation of peripheral antenna activity,^85^, ^88^, ^89^ which is not in the focus of the present review, interesting issues concerning light harvesting are (a) the pigment composition of the peripheral antenna, (b) the supramolecular organization of the complexes in the thylakoid membrane, and (c) the differences between species in the structure and composition of the core complex.

To extend the spectrum of photons that can be absorbed by the antenna system, plants incorporate besides chlorophyll (Chl) *a* the slightly modified Chl *b* in their peripheral antennae, which is why they have been referred to as chlorophyll *a*/*b* binding proteins formerly. Nowadays, one distinguishes between the trimeric LHCII, of which there are two copies, M‐LHCII and S‐LHCII, in each monomer of the C_2_S_2_M_2_ supercomplex, and the monomeric homologs CP24, CP26, and CP29 (Figure 3a). In addition, there are homologous PPCs attached to PSI.^8^, ^9^, ^11^ The broadening of the absorption spectrum of the antenna is due to the absorption maximum of Chl *b* being blue‐shifted by about 20 nm with respect to that of Chl *a* because of the substitution of a methyl group at ring B with a formyl group.^1^, ^90^

Diatoms are marine organisms that evolved from red algae by secondary endosymbiosis^91^, ^92^ and are responsible for ~40% of the net primary production^91^ (i.e., the formation of organic material from inorganic compounds minus the respiratory losses^93^). They are characterized by a siliceous cell wall called *frustule*, which is an interesting topic in itself^94^ and has triggered research in biomimetic nanotechnology.^95^, ^96^, ^97^ In contrast to plants, diatoms incorporate Chl *c* in their peripheral antennae.^98^ Unlike Chls *a* and *b* that are chlorins with ring D saturated, Chl *c* is actually a porphyrin with four unsaturated pyrrole rings.^1^, ^90^ As a consequence, its major absorption is in the Soret region around 450 nm. Another difference to the plant system is the high amount of the carotenoid fucoxanthin in the peripheral antenna of diatoms giving rise to the name fucoxanthin‐chlorophyll *a*/*c* binding proteins (FCPs) for this type of peripheral antenna. Depending on whether the complexes are associated with PSI or PSII, they are termed FCPI or FCPII, respectively. As can be seen from Figure 3b, there are monomeric variants (FCP‐D, FCP‐E, and FCP‐F) and tetramers (MT and ST) in the PSII‐FCPII supercomplex.

A characteristic feature of the thylakoid membrane in plant chloroplasts is its extensive folding resulting in basically two different domains: the grana, which appear as stacks of flat, pancake‐like membrane sheets enclosing the lumen and sticking to each other with the stromal surface, and stroma lamellae, which are unstacked and seem to connect the grana.^22^, ^86^, ^87^ EM investigations of grana membranes revealed crystalline two‐dimensional arrangements of PSII complexes that might be composed of certain types of supercomplexes identified in single‐particle analyses. Note that the C_2_S_2_M_2_ supercomplex, of which the cryo‐EM structure is now known at high resolution (Figure 3a), is not the only type of supercomplex observed. The designations “S” and “M,” with which the various LHCII copies in the supercomplex are labeled, refer to “strongly” and “moderately” bound, respectively. Accordingly, for example, an M‐LHCII might be lost during preparation or dissociate off in the membrane, resulting in a C_2_S_2_M complex. In addition, further copies of LHCII can be more “loosely” attached to the supercomplex (and consequently are termed L‐LHCII). Originally, it was unclear, whether the semicrystalline arrays of PSII supercomplexes represent a native or functional state of the membrane.^86^ There is evidence, however, that the crystalline arrays are of physiological relevance,^87^, ^99^, ^100^, ^101^, ^102^ supporting the idea that a regular superstructure is of advantage for an efficient light‐harvesting under low‐light conditions. In addition, it has been suggested that the arrays facilitate PQ diffusion.^86^, ^103^

Very recently, information about the spatial organization of proteins in membranes of the diatom *Phaeodactylum tricornutum* was obtained.^104^ Two subpopulations of PSII complexes were identified. One consists of irregular clusters of supercomplexes, in which dPSIIcc is associated with FCPs (apparently with less FCPs than in the supercomplex from *Chaetoceros gracilis* shown in Figure 3b). The second subpopulation appears as two‐dimensional crystalline arrays of dPSIIcc and was suggested to represent reservoirs of photodamaged PSII. This interpretation throws a new idea into the discussion about the functional role of these arrays.

Figures 2 and 3 illustrate an important point: Although dPSIIcc is largely conserved, it has different environments in the various organisms. These variations may have caused adaptations of dPSIIcc in evolution with consequences for subunit composition and pigment energy levels.

## 3 SUBUNITS OF THE CORE COMPLEX

**Figure 4:**
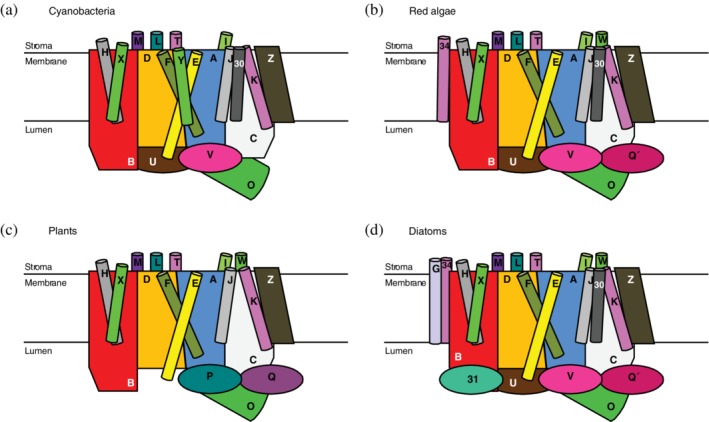
Schematic representation of the protein subunit composition of PSIIcc in various organisms as inferred from X‐ray and cryo‐EM structures (cf. Table 1). The subunits are labeled according to the “Psb” nomenclature

The core complex of PSII is composed of a large number of membrane‐intrinsic and (at least) three membrane‐extrinsic protein subunits encoded by *psb* genes and labeled with the prefix “Psb.” In addition to the structural proteins identified in the crystal and EM structures, there are auxiliary proteins, which are involved in the biogenesis and maintenance of PSII. The proteins (and genes) discovered earlier were labeled with capital letters (i.e., PsbA, PsbB, etc., but there are deviations from this simple scheme to be discussed below). However, since there are more than 26 of them, the later ones got numbers (e.g., Psb30). Some subunits have alternative traditional names (e.g., D1 protein for PsbA). A comprehensive list (as of 2013) is given by Pagliano et al.^105^ The current picture based on structural information (i.e., excluding auxiliary subunits) is listed in Table 1 and expressionistically illustrated in Figure 4.

The major part of the PSII core complex is constituted by the four large membrane‐intrinsic subunits PsbA, B, C, D, with the two central subunits PsbA (D1) and PsbD (D2) forming a pseudo C_2_‐symmetric heterodimer and interlocking in a handshake motif homologous to the purple bacterial reaction center (PBRC).^106^, ^107^ The D1‐D2‐heterodimer harbors almost all redox‐active cofactors of PSIIcc including the RC (Figure 1; more details below) and is highly conserved. For completeness, we should mention that there are several PsbA isoforms in cyanobacteria, the role of which has not yet finally been clarified.^108^, ^109^

Pursuing the pseudo symmetry, the other two large subunits PsbB (CP47) and PsbC (CP43) are attached to both sides of the RC part (Figure 2a) and establish the core antennae.^13^ However, PsbB and C apparently serve more purposes than just holding the core antenna pigments in place within the membrane, as they have significant extensions into the lumen, where they are in close contact with the membrane‐extrinsic subunits (Figure 2b). PsbC even provides a direct ligand to metal ions of the WOC (Glu C354),^19^ but the role of this arrangement remains obscure.

Structures, properties, and putative functions of the extrinsic subunits have been reviewed.^110^, ^111^, ^112^, ^113^, ^114^ In the context of the present review, the important aspect is the interaction of these subunits with the large membrane‐intrinsic subunits. According to the crystals structures, cyanobacterial PSIIcc contains the extrinsic subunits PsbO, U, and V (Figure 4a). PsbO, also referred to as 33 kDa protein or manganese‐stabilizing protein, is highly conserved among species as regards overall structure and position within PSIIcc. Functionally, PsbO stabilizes the Mn_4_CaO_5_ cluster under suboptimal chloride concentrations and may also assist in maintaining the association of Ca^2+^ with the WOC.^111^ It has also been suggested to play a role in proton transfer away from the WOC.^115^, ^116^, ^117^, ^118^, ^119^ Since high proton concentrations in the vicinity of the WOC are counterproductive in the water splitting process, a continuous removal of protons is required. PsbO is in contact with PsbB, C, and D involving titratable groups^116^ and has been suggested to undergo pH‐dependent conformational changes.^120^ The latter are local^117^ and probably do not affect the overall PSIIcc structure. The high conservation of accessible, negatively charged surface residues in PsbO suggests an additional function as local pH buffer or proton antenna.^115^, ^118^, ^121^ In a proton antenna, the surface carboxylate groups that are located close to each other might retain a proton for longer times than a single group. A pH buffer functionality of PsbO could transiently avoid acidification of the thylakoid lumen for conditions of fluctuating light intensities (but not under continuous illumination) as they easily and often occur in a natural habitat. This process may be relevant in a time scale of 5–20 s,^122^ which is a typical time regime for luminal acidification after an increase of light intensity in intact organisms. In cyanobacteria, PsbO features a specific loop not found in other organisms, coined the “cyano loop,”^123^ that mediates inter‐dimer contact in the rows shown in Figure 2a.^37^ Insofar as the rows may influence light‐harvesting as discussed above, PsbO could play a structural role here, but this is certainly only a side aspect of its function.

PsbV and U occur in cyanobacteria, red algae, and diatoms, but not in plants (Figure 4). However, in the latter, PsbV is replaced with PsbP. Despite the fact that PsbV is a heme protein, also referred to as cytochrome *c*_550_, and PsbP is not, both seem to have analogous functions in that they maintain the affinity of PSII for Ca^2+^ and Cl^−^ to support integrity of the WOC.^110^, ^124^ Accordingly, the role of the heme remains elusive. We note that PsbP has a cyanobacterial counterpart, CyanoP,^110^, ^111^, ^125^ that is not seen in the structures, so that it is unclear, where and how it binds to PSIIcc. Plant PSIIcc binds another extrinsic protein, PsbQ, which has counterparts denoted as PsbQ´ in read algae and diatoms, but not in cyanobacteria. However, the latter contain a CyanoQ not seen in the PSIIcc‐structures, but placed by docking studies in a position next to PsbV similar to PsbQ´.^125^ Finally, diatoms feature a hitherto unobserved subunit Psb31, which is structurally similar to PsbQ, but bound at a different position next to CP47 and D2.

When overlaying all the PSIIcc‐structures from the different organism types, it appears that the overall structure of the core complex is less variable than suggested by the multitude of extrinsic proteins, and in particular the membrane‐spanning part containing the chlorophylls remains largely unaffected by the diversity of the luminal domain despite the contact of CP43 and CP47 with the extrinsic subunits. This impression is in line with our hypothesis, to be further discussed below, that the antenna‐RC part of the PSIIcc‐structure is not strongly influenced by the water oxidation action supported by the extrinsic proteins in order to guarantee light‐harvesting and charge separation (CS) in all catalytic steps of the WOC.

The small membrane‐intrinsic subunits (SMIS) have been described in reviews.^105^, ^126^, ^127^ In the present review, we focus on certain aspects: (a) the denomination and assignment of SMIS, in particular in the new structures, (b) the possible loss of SMIS during crystallization, (c) the crystallographic characterization of a deletion mutant, and (d) the interaction of SMIS with the antenna subunits CP43 and CP47. We note that the SMIS have been named low molecular weight subunits in the past and today are commonly designated low molecular mass subunits. Here, we use the term SMIS to underscore that they are not only small but also membrane spanning. A particular SMIS is preferably referred to by using its “Psb” letter or number. According to the crystal structures, cyanobacterial PSIIcc contains the SMIS PsbE, F, H, I, J, K, L, M, T, X, Y, and Z as well as Psb30 (Table 1). This list demands some comments: PsbE and F are particular in that they bind a heme group and thus constitute the α‐ and β‐subunit, respectively, of cytochrome *b*_559_. Like its membrane‐extrinsic heme‐binding counterpart in cyanobacteria, red algae and diatoms, cyt *c*_550_ (PsbV, see above), cyt *b*_559_ has no function that could so far unambiguously be assigned to it.^29^ PsbY is closely attached to PsbE/F^29^ and could be considered part of cyt *b*_559_, if it were not apparently absent in all but the cyanobacterial lineage. However, a *psbY* gene is found in algae and plants^105^ suggesting that PsbY is either not permanently bound to PSIIcc or lost in the biochemical preparation. Indeed, PsbY is not seen in all crystal structures of cyanobacterial PSIIcc or partly lost (i.e., in one monomer of dPSIIcc) for reasons discussed below. PsbZ has the specific property of featuring two trans‐membrane helices (TMHs), whereas all other SMIS possess only one. Psb30 is also known as Ycf12 and therefore referred to in the crystal structures as “subunit Y,” which may cause confusion. In fact, PsbY is called “subunit R.”

**Figure 5:**
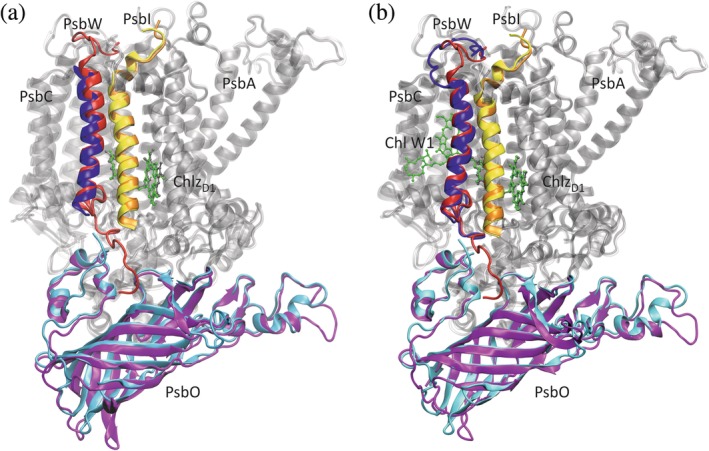
Overlay of PSIIcc structures, where the respective PsbA subunits were aligned using MultiSeq.^130^ The protein backbone is shown in new cartoon mode, Chls in CPK mode. (a) Plant (Chlz_D1_, gray; PsbA/C, gray; PsbI, orange; PsbO, cyan; PsbW, red) and red alga (Chlz_D1_, green; PsbA/C, white; PsbI, yellow; PsbO, magenta; “subunit S,” blue). (b) Plant (Chlz_D1_, gray; PsbA/C, gray; PsbI, orange; PsbO, cyan; PsbW, red) and diatom (Chlz_D1_ and Chl W1 green; PsbA/C, white; PsbI, yellow; PsbO, magenta; PsbW, blue). Figure made with VMD^32^ based on PDB ID 5XNL,^46^ 4YUU,^39^ and 6JLU^48^

The naming in PDB‐files is an even more severe problem in the PSIIcc‐structures of the other lineages, and one has to watch out in order not to mix up the subunits. Another possible reason for puzzlement is the fact that eukaryotic organisms contain a second “PsbT” protein. The SMIS PsbT is chloroplast‐encoded and therefore often referred to as PsbT_c_. In addition, there is a nuclear‐encoded PsbT_n_ that is not a SMIS but an extrinsic subunit^105^ identified in the “unstacked” C_2_S_2_M_2_ supercomplex from plants (not shown in Figure 4). The present analysis is based on the “stacked” supercomplex (not containing PsbT_n_) as it features a higher resolution.^46^ The latter plant structure does not contain PsbY as mentioned above, but shows an additional SMIS called PsbW (Figure 4c). The corresponding gene *psbW* is not found in cyanobacteria. PsbW is nuclear‐encoded and binds to CP43 in close contact with PsbI, but without any obvious direct interaction with the tetrapyrrole system of a Chl. The N‐terminus of PsbW is located in the lumen in accordance with predictions,^128^, ^129^ where it interacts with PsbO (Figure 5a).

**Figure 6:**
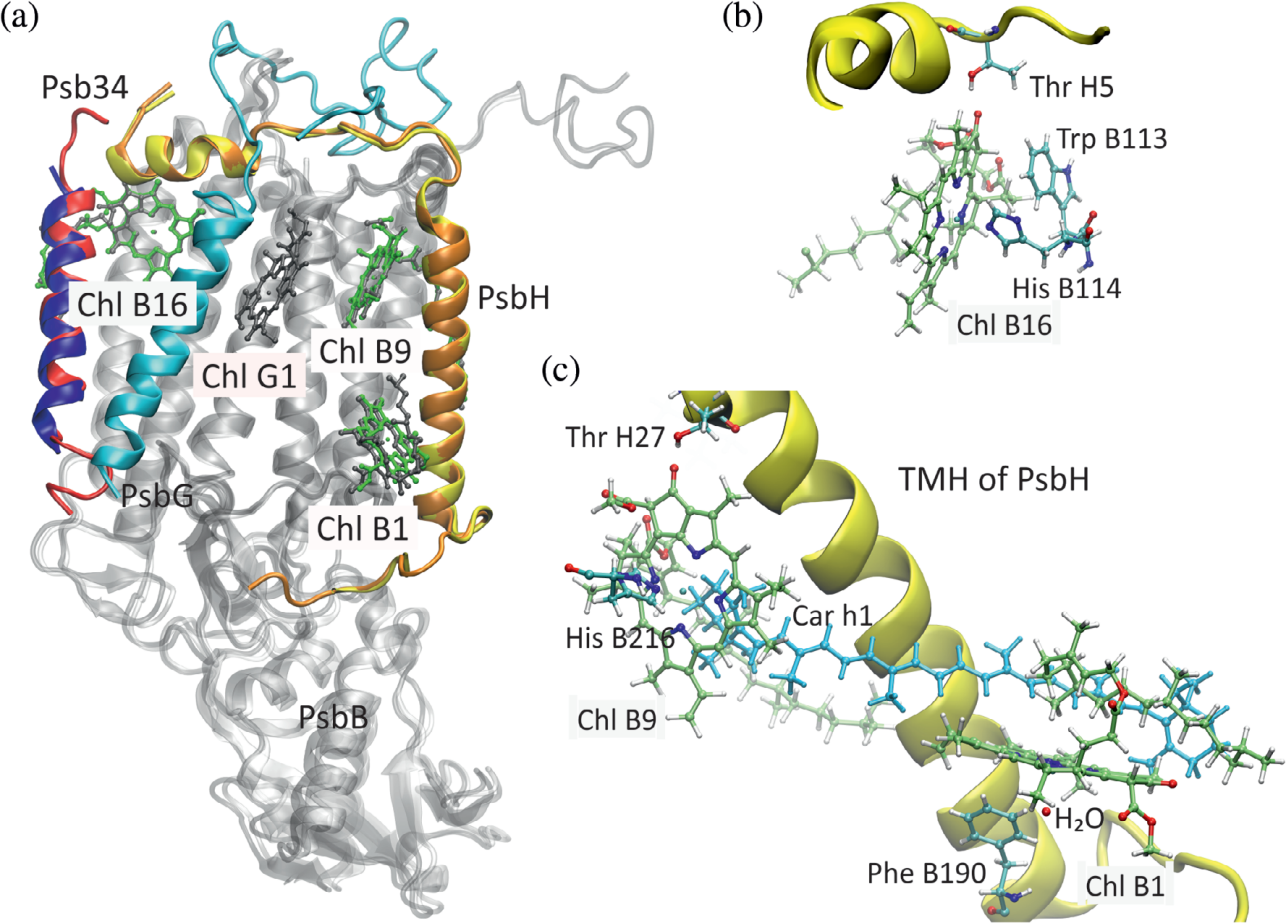
(a) Overlay of PSIIcc structures from diatoms (Chls, gray; PsbB, gray; PsbH, orange; Psb34, red; PsbG, cyan) and red algae (Chls, green; PsbB, white; PsbH, yellow; “subunit W,” blue), where the respective PsbA subunits were aligned using MultiSeq.^130^ The protein backbone is shown in new cartoon mode, Chls in CPK mode. (b) Binding site of Chl B16 in cyanobacteria close to the N‐terminal short helix of PsbH, (c) Binding sites of Chls B9 and B1 in cyanobacteria along the TMH of PsbH connected by a β‐carotene pigment (Car h1, light blue; cf. Table 3). The small red sphere close to Chl B1 represents the oxygen atom of the axially ligating water molecule (H_2_O). Figure made with VMD^32^ based on PDB ID 4YUU,^39^ 6JLU,^48^ and 6DHE^26^

The structure of PSIIcc from red algae also lacks PsbY, but contains two additional SMIS denoted “subunit S” and “subunit W.”^39^ A comparison with the plant structure suggests that “subunit S” very likely is actually PsbW or a red alga homolog of it (Figure 5a) and is labeled as such in Figure 4b. Similarly, a comparison with the diatom structure suggests that “subunit W” is actually Psb34 (Figure 4b,d), a SMIS not observed before.^48^ This subunit comes close to Chl B16 of CP47 (Figure 6a). PSIIcc from diatoms as apparent from the structure lacks PsbY, but contains PsbW, Psb34, and a new SMIS called PsbG (Figures 4d and 6a). A peculiar feature of the diatom structure is that some SMIS (i.e., PsbG, W, and Z) bind additional Chls not present in the other lineages (Table 2). The Chls are discussed below.

**Table 2:** Chlorin pigments in the photosystem II core complex with numbering schemes, characteristic features of their protein environment, and modeled site energies

#^a^	2AXT^b^	3ARC^c^	6DHE^d^	4YUU^e^	5XNL^f^	6JLU^g^	Axial^h^	Keto^i^	PBQC^j^	CDC^k^
P_D1_	P_D1_	604	A 404	A 403	A 405	A 403	His A198	–	668	663
P_D2_	P_D2_	605	D 403	D 402	D 402	D 404	His D197^l^	–	668	654
Chl_D1_	Chl_D1_	606	A 411	A 406	A 406	D 401	H_2_O	H_2_O	**678**	**682**
Chl_D2_	Chl_D2_	607	A 405	A 404	A 407	A 404	H_2_O	H_2_O	670	670
Pheo_D1_	Pheo_D1_	608	A 406	A 408	A 408	A 405	–	Gln A130^m^	670	663
Pheo_D2_	Pheo_D2_	609	D 402	D 407	A 409	D 402	–	Gln D129^n^	675	669
Chlz_D1_	Chlz_D1_	610	A 407	A 405	A 410	A 406	His A118	Ile A96 (B)	670	675
Chlz_D2_	Chlz_D2_	611	D 404	D 403	D 403	D 405	His D117^o^	Leu D92 (B)^p^	665	674
B1	11	612	B 601	B 604	B 602	B 601	H_2_O	–	**688**	676
B2	12	613	B 602	B 605	B 603	B 602	His B201	–	665	662
B3	13	614	B 603	B 606	B 604	B 603	His B202	Arg B68	672	674
B4	14	615	B 604	B 607	B 605	B 604	His B455	–	672	667
B5	15	616	B 605	B 608	B 606	B 605	His B100	–	662	665
B6	16	617	B 606	B 609	B 607	B 606	His B157	–q	676	671
B7	17	618	B 607	B 619	B 608	B 607	H_2_O	Tyr B40^r^	676	674
B8	21	619	B 608	B 610	B 609	B 608	His B466	H_2_O	668	668
B9	22	620	B 609	B 611	B 610	B 609	His B216	**Thr H27** s	669	668
B10	23	621	B 610	B 612	B 611	B 610	H_2_O	His B142	667	672
B11	24	622	B 611	B 613	B 612	B 611	His B469	H_2_O	**683**	664
B12	25	623	B 612	B 614	B 613	B 612	His B23	Ser B241	663	675
B13	26	624	B 613	B 615	B 614	B 613	His B26	–	669	669
B14	27	625	B 614	B 616	B 615	B 614	His B9	–t	**679**	667
B15	28	626	B 615	B 617	B 616	B 615	His B142	His B23	670	673
B16	29	627	B 616	B 618	B 617	B 616	His B114	**Thr H5** u	677	676
C1	33	628	C 501	C 502	C 501	C 502	His C237	H_2_O	668	671
C2	34	629	C 502	C 503	C 502	C 503	His C430	–	660	672
C3	35	630	C 503	C 504	C 503	C 504	His C118	–	676	670
C4	37	631	C 504	C 505	C 504	C 505	H_2_O	**LMG C519** v	**681**	669
C5	41	632	C 505	C 506	C 505	C 506	His C441	H_2_O	667	670
C6	42	633	C 506	C 507	C 506	C 507	His C251	–	669	666
C7	43	634	C 507	C 508	C 507	C 508	H_2_O	His C164	677	669
C8	44	635	C 508	C 509	C 508	C 509	His C444	–	671	665
C9	45	636	C 509	C 510	C 509	C 510	His C53	Ser C275	677	674
C10	46	637	C 510	C 511	C 510	C 511	His C56	–	670	670
C11	47	638	C 511	C 513	C 511	C 512	Asn C39	Arg C41	677	668
C12	48	639	C 512	C 512	C 512	C 513	His C164	H_2_O	673	673
C13	49	640	C 513	C 514	C 513	C 514	His C132	–w	669	667
G1	–	–	–	–	–	R 101	‐	–	–	–
W1	–	–	–	–	–	V 202	Glu W113	Trp W114	–	–
Z1	–	–	–	–	–	Y 101	Ala Z55^x^	–	–	–

a Recommended numbering.

b Traditional numbering.

c Previous PDB numbering *Th. vulcanus*.

d PDB numbering *Th. elongatus* (monomer with capital letters designating subunits).

e PDB numbering *C. caldarium* (monomer 1 with capital letters designating subunits).

f PDB numbering *P. sativum* (monomer with capital letters designating subunits).

g PDB numbering *Ch. gracilis* (monomer with capital letters designating subunits).

h Axial ligand.

i Hydrogen‐bond donor to 13^1^‐keto group; “B” indicates hydrogen bond from the polypeptide backbone; bold letters indicate hydrogen bond donors that are neither water molecules nor amino acid residues from PsbA, B, C or D (based on 6DHE).

j Site energy (converted to the nm scale) computed with the PBQC method and refined by comparison with experiment (Müh et al.,^28^, ^131^ Hall et al.^132^); bold numbers indicate strongly red‐shifted site energies (≥678 nm).

k Site energy (converted to the nm scale) computed with the CDC method based on MD trajectories and averaged over the two monomers (Hsieh et al.^133^); bold numbers indicate strongly red‐shifted site energies (≥678 nm).

l His D198 in 5XNL.

m Glu A130 in 4YUU, 5XNL, and 6JLU.

n Gln D130 in 5XNL.

o His D118 in 5XNL.

p Leu D93 (B) in 5XNL.

q LMG B622 in 4YUU; LHG B2631 in 5XNL; LMG N101 in 6JLU.

r LMG B620 in 6JLU.

s Thr H39 in 5XNL and 6JLU.

t H_2_O in 5XNL; LMG M101 in 6JLU.

u Thr H17 in 5XNL and 6JLU.

v LMG C520 in 4YUU; LMG C521 in 5XNL; LMG K101 in 6JLU.

w LMG Y102 in 6JLU.

x Backbone, probably via H_2_O.

PsbY is not observed in all cyanobacterial dPSIIcc structures. Of particular interest are the structures 4IL6,^36^ 4UB6, and 4UB8,^38^ as well as 5GTH, 5GTI, 5WS5, and 5WS6.^42^ In these structures, PsbY is only present in one monomer of dPSIIcc. This finding suggests that SMIS might be lost during the crystallization process due to packing constraints in the crystal. A similar observation was made with PsbZ in the red alga structure 4YUU, where PsbZ is only present in two of the four monomers.^39^ It has been suggested that PsbY and PsbZ are only weakly bound to the core complex and, therefore, easily lost.^39^ We would like to add that the binding of hydrophobic, membrane‐spanning peptides is certainly influenced by the detergent used to solubilize PSIIcc. The detergent type, the absolute detergent concentration, and the detergent‐protein ratio are probably influential in this regard^60^, ^61^ and deserve a more systematic investigation in the future.

The major membrane‐intrinsic peptide contact between the monomers in dPSIIcc is provided by the two copies of PsbM, who interact by virtue of a heptad motif of aliphatic side chains as in a leucine zipper.^56^ Accordingly, it has been suggested that PsbM is important, but not essential for dimer formation. The predictions were confirmed by a crystallographic study of a PsbM‐deletion mutant (ΔPsbM‐PSII).^43^ Dimers are still formed, but destabilized. The lack of PsbM causes some reorganization of the surrounding protein structure as well as protein‐bound lipids with consequences for the ET from Q_A_ to Q_B_.^43^ Possible effects of these structural perturbations on light harvesting remain to be elucidated.

The SMIS that is probably of highest interest in the context of light harvesting is PsbH, which binds to CP47 and is in contact with three Chls (Figure 6) that may contribute to the red‐shifted states of the core antenna (see below). Accordingly, we shall have a closer look on the pigments of the core complex next.

## 4 PIGMENTS AND LIPIDS OF THE CORE COMPLEX

PSIIcc binds 35 Chl *a* and two Pheo *a* pigments, where Pheo is the free‐base variant of Chl (i.e., it contains two protons instead of the central Mg^2+^ ion^1^, ^13^). Notably, PSIIcc of diatoms features three additional Chl *a* pigments associated with SMIS. All the chlorin pigments can be assigned to a protein subunit based on their location and/or the origin of the axial (fifth) ligand to the Mg^2+^ ion (Table 2). Another characteristic of the protein environment is the hydrogen bond donor to the 13^1^‐keto group if present. These indicators are very helpful for correctly assigning homologous pigment sites in the various structures given that the pigment numbering in the PDB files is not unequivocal as can be seen from the examples listed in Table 2. To minimize confusion in future work, we recommend using the pigment labels suggested by us in the first column of Table 2. These labels employ the traditional naming of pigments bound to the D1‐ and D2‐proteins (see also Figure 1) and a consecutive subunit‐specific numbering of antenna pigments based on the prevailing order of Chls in the earlier structure files.^13^ For comparison, we also give the traditional numbering of antenna Chls based on the work of Loll et al.^33^ (denoted 2AXT) and the numbering scheme due to Umena et al.^35^ (denoted 3ARC), since these are frequently used in the literature.

Four Chls (P_D1_, P_D2_, Chl_D1_, and Chl_D2_) and the two Pheos (Pheo_D1_ and Pheo_D2_) belong to the RC (Figure 1) and are at the interface between EET and CS as further discussed below. The two extra Chls, Chlz_D1_, and Chlz_D2_ bound to PsbA and PsbD, respectively, do not belong to the RC. Their function is unclear, but Chlz_D2_ may be involved in slow ET reactions associated with cyt *b*_559_.^29^, ^134^

The Chls bound to PsbB (CP47) and PsbC (CP43) are arranged in a way that two main layers of pigments result, a stromal and a luminal layer, while only two Chls (B13, C10) are located in the middle of the membrane slab. Pictorial representations of the layer structure and further details concerning Chl‐protein interactions can be found in our earlier review.^13^ Here, we focus on (a) the three Chls of CP47 (B1, B9, and B16) interacting with PsbH (Figure 6), (b) the sites of Chls B7 and C4, which are particular and underwent a significant structural refinement compared to the structure at a resolution of 3 Å, and (c) the three newly discovered Chls G1, W1, and Z1 in the supercomplex structure of diatoms. These cases will also give us the opportunity to discuss carotenoids and lipids bound to PSIIcc.

To understand the significance of PsbH in the context of light‐harvesting, we have to consider low‐energy states that have been discovered in many photosynthetic systems as reviewed by Reimers et al.^135^ These states become manifest in optical absorptions energetically well below that of the RC, that is, in the case of PSII at wavelengths larger than about 680 nm (see also the discussion of the RC absorption spectrum below). A distinction has to be made between extremely red‐shifted states of yet unknown origin^135^ and more moderately red‐shifted states giving rise to absorption at 694 nm as well as corresponding emission signals.^132^, ^136^, ^137^, ^138^, ^139^, ^140^, ^141^, ^142^, ^143^ We are only concerned with the latter type of states here. Since excitation energy arriving at these states has to go energetically uphill to reach the RC, they cause a complicated temperature dependence of the fluorescence of dPSIIcc as explained in detail by Shibata et al.^138^ The pigments being responsible for the low‐energy states are often referred to in a somewhat sloppy way as “red Chls,” and it is consensus that they belong to CP47. This assignment is based on the observation that red‐shifted spectral features are also found in isolated CP47, whereas isolated CP43 and RCs do not show them. It should be noted that subcomplexes of PSIIcc can be purified from plant material including CP43,^144^, ^145^, ^146^ CP47,^146^, ^147^, ^148^ and a D1‐D2‐cyt‐*b*_559_ complex that contains PsbI besides PsbA, D, E, and F.^149^, ^150^, ^151^ Subcomplex preparations based on material from other organisms have also been reported such as an RC from the green alga *Chlamydomonas reinhardtii* 152, 153 and His‐tagged CP43 and CP47 from the mesophilic cyanobacterium *Synechocystis* sp. PCC 6803.^154^ The latter work by Boehm et al.^154^ is of particular interest as it demonstrates that in the assembly of PSIIcc, CP43 and CP47 are preassembled and bind pigments and SMIS before being integrated into the core complex (assembly and repair cycle of PSII have been reviewed).^155^, ^156^ This result enabled a spectroscopic investigation of a complex containing CP47 as well as PsbH, L, and T (the SMIS probably in substoichiometric amounts) and a comparison with standard CP47 preparations from spinach obtained by disintegration of core complexes. The peptide composition of the latter is actually not clear, but it is widely assumed that they do not contain SMIS. The absorption and fluorescence spectra indicated the presence of red Chls in both types of samples, but less pronounced in the cyanobacterial preparations. More recently, D'Haene et al.^157^ investigated a PsbH‐deletion mutant of *Synechocystis* (against a PSI‐deficient background). They confirmed the (relative to spinach) less pronounced absorption and emission bands of red Chls in intact core complexes as well as in an assembly intermediate (containing besides CP47 also PsbH, L, M, and T) and showed that these red‐shifted spectral features were further reduced, if not eliminated, in the absence of PsbH. These results allow for two conclusions: (a) PsbH affects the low‐energy states in that it causes the red‐shift of at least part of the Chls involved, and (b) there are significant differences between cyanobacteria and plants as the red emission is more pronounced in the latter, probably even in the absence of PsbH. A problem is that disintegration of the core complex may damage CP47. Indeed, Jankowiak and coworkers argue on the basis of data from hole‐burning spectroscopy^158^ that the spinach samples are heterogeneous and possibly not intact.^142^, ^159^ Thus, it remains unclear at present, to what extent the differences between cyanobacteria and plants are due to PsbH, species‐specific differences in PsbB or artifacts of biochemical preparation procedures.

PsbB and PsbH as well as their mutual interaction and their interactions with Chls are highly conserved between lineages. Irrespective of differences in the amino acid sequence, the consequences of which remain to be analyzed in detail, PsbH has one TMH that lies at the membrane‐facing surface of PsbB close to Chls B1, B2, and B9. The N‐terminal part of PsbH features a long arm that reaches out at the stromal surface to the other side of PsbB (Figure 6a) attaining the monomer–monomer interface of dPSIIcc with a short helical segment forming part of the binding sites of Chl B15 and B16. Based on the experimental results described above, it is reasonable to search for the red Chls among those that are in contact with PsbH. Of these, B9 and B16 accept hydrogen bonds from threonine residues of PsbH and are thus the only Chls having such a kind of interaction with a SMIS (Table 2). Together with B1, they are of particular interest as discussed further below after some theoretical considerations.

These Chls are also in contact with carotenoids. PSIIcc features 11 β‐carotene (Car) molecules per monomer (Table 3). Of these, one is missing in the structures of red algae and diatoms, but is also missing in some of the cyanobacterial structures. A 12th Car assigned in the structure at 2.9 Å resolution (close to Car_D2_)^34^, ^56^ could not be found in any of the other structures and, therefore, is not listed in Table 3. As with the Chls, the PDB numbering of Cars is not unequivocal, and we propose a unified numbering scheme in the first column of Table 3. All Car are found in the periphery of the PSIIcc‐monomer,^13^ implying that some are located at the monomer–monomer interface in dPSIIcc or between TMHs of the large membrane‐intrinsic subunits and SMIS. Only two Car are structurally related to the RC subunits PsbA and PsbD. We propose that these two Car should be referred to by their traditional names Car_D1_ and Car_D2_, not least because they are in close contact with Chlz_D1_ and Chlz_D2_, respectively (Figure 1). As with Chlz_D2_, Car_D2_ may be involved in redox reactions.^29^, ^134^, ^137^

**Table 3:** Carotenoids in the photosystem II core complex with numbering schemes and close contacts to chlorophylls

#^a^	6DHE^b^	4YUU^c^	5XNL^d^	6JLU^e^	Nearby Chl: Shortest π‐π edge‐to‐edge distance in Å^f^
Car_D1_	A 408	A 401	A 411	A 407	Chlz_D1_: 4.2 (Figure 1)
Car_D2_	D 404	D 401	D 404	F 101	Chlz_D2_: 6.6 (Figure 1)
b1	B 617	B 601	B 618	B 617	B7: 6.2, B13: 5.1, B14: 3.8 (3‐vinyl^g^)
b2	B 618	B 602	B 619	B 618	B4: 5.4 (3‐vinyl^g^), B7: 4.6, B13: 4.8
b3	B 619	B 603	B 620	B 619	B5: 4.6 (3‐vinyl^g^), B16: 4.4
c1	C 514	J 101^h^	C 514	C 516	C12: 6.0, C13: 4.0, W1: 4.4^i^
c2	C 515	C 501	C 515	C 515	C1: 4.2 (3‐vinyl^g^), C5: 5.0, C7: 4.4 (3‐vinyl^g^), Z1: 4.7^i^
c3	C 520	C 521	C 517	C 517	C11: 4.1
h1	H 101	G 102^h^	H 101	H 101	B1: 3.6 (Figure 6c), B2: 5.6, B9: 3.8 (Figure 6c), B10: 6.6 (3‐vinyl^g^)
j1	Y 101	I 101	C 516	X 101	–
t1	T 101	–	T 101	–	B7′ (of the second monomer in dPSIIcc): 5.1

a Recommended numbering.

b PDB numbering *Th. elongatus* (monomer with capital letters designating subunits).

c PDB numbering *C. caldarium* (monomer 1 with capital letters designating subunits).

d PDB numbering *P. sativum* (monomer with capital letters designating subunits).

e PDB numbering *Ch. gracilis* (monomer with capital letters designating subunits).

f Based on 6DHE.

g Via 3‐vinyl group of Chl.

h Incompletely modeled.

i Only in diatoms.

Of the remaining Car pigments, three are assigned to PsbB and hence labeled with a lower‐case b (to avoid confusion with the numbering of Chls employing capital letters), and similarly three Car are assigned to PsbC. Another Car is located between PsbB and the TMH of PsbH and therefore is labeled h1 (Figure 6c). The 10th Car is associated with Psb30, which is also referred to as “subunit Y.” To avoid confusion, we consider the vicinity of this Car to PsbJ and call it j1. Finally, there is a Car next to PsbT labeled t1. This Car has the special property of crossing the monomer–monomer interface in dPSIIcc. It could not be detected in the structure of monomeric PSIIcc,^67^ which can be rationalized on the basis of its position. However, Car t1 is also missing in many dPSIIcc‐structures including those of red algae and the diatom supercomplex (Table 3) for unknown reasons.

Carotenoids have several essential functions in photosynthesis,^1^ two of which are interesting for PSIIcc: (a) They are accessory pigments for light‐harvesting, absorbing light and transferring the energy to Chls, and (b) they rapidly quench triplet states of Chls to prevent formation of singlet oxygen. The second function is probably the more important one in PSIIcc, but the first will also play a role further below in the context of investigating EET.

Chlorophylls have a rather high triplet yield, which is, however, strongly dependent on solvent and temperature.^160^, ^161^ In the protein environment of PSII, the triplet yield is high enough to necessitate the presence of carotenoids as quenchers.^162^ Since triplet–triplet energy transfer (TTET) from Chl to Car requires electron exchange between the two pigments, the π‐systems of both cofactors have to approach each other to a distance of less than about 5–6 Å, and probably to van der Waals distance (below 4 Å) for very efficient TTET.^163^ It should be noted that TTET is a kind of double ET process, whose distance dependence can be modeled in the simplest approach similar to the Moser–Dutton ruler for ET.^164^ However, the exponential attenuation factor of the electronic coupling is larger for TTET than for ET (by a factor of about 2), that is, closer distances are required for TTET.^163^, ^165^, ^166^ As can be seen from Table 3, almost all Car in PSIIcc are close to at least one Chl. The distances given in Table 3 are derived from structure file 6DHE^26^ obtained with RT SFX and thus refer to nearly physiological temperature conditions, which has an influence on the distances.^26^, ^27^ Notably, only three Chls (B1, B9, and B14, all in CP47) are literally in van der Waals contact with a Car as regards the π‐systems. Of particular interest for the further discussion of red Chls below is the van der Waals contact of Car h1 (bound to PsbH) with Chls B1 and B9 (Figure 6c). Other Chls approach Car still to less than 4.5 Å including B16 interacting with Car b3 (not shown in Figure 6). Since the formation of the Chl triplet state requires some time and the precursor singlet excited state is trapped at sites with low energy (or low‐energy exciton states, see below), triplets are preferentially formed at such sites, and triplet quenchers should be positioned there. Thus, another criterion in the quest for red Chls is the contact with Car.

**Figure 7:**
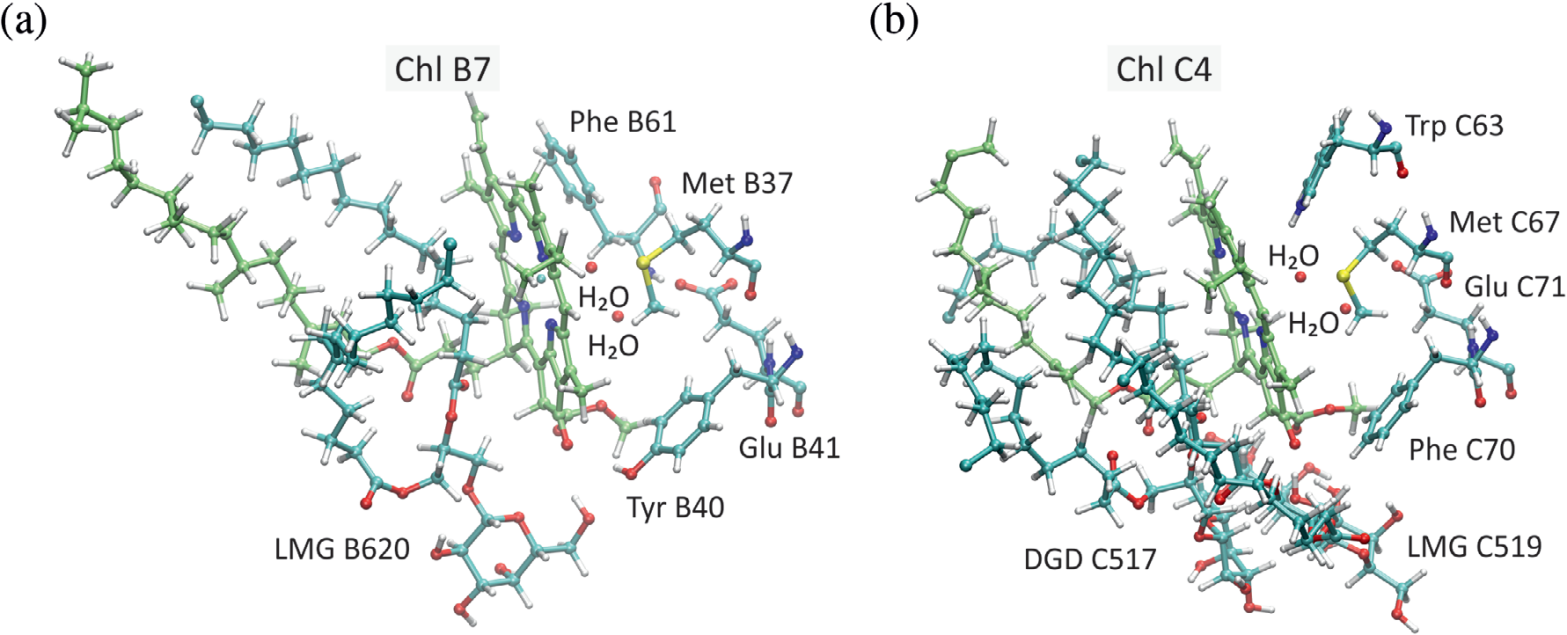
Binding sites of Chls B7 (a) and C4 (b) based on PDB ID 6DHE^26^ (Chls with carbon green; LMG = MGDG: monogalactosyldiacylglycerol; DGD = DGDG: digalactosyldiacylglycerol). Figures made with VMD^32^

The two Chls B7 and C4 are located in symmetry‐related positions in CP47 and CP43, respectively. Their binding sites are similar, but distinct from those of other Chls in several respects. The axial ligand is now clearly identified as a water molecule. While this type of ligand is not a special property (cf. Table 2), the water ligand in these sites is hydrogen‐bonded to a second water molecule, which in turn is bound to a Glu side chain (Figure 7). Based on electrostatic computations, this Glu can be considered to be in a standard protonation state, which is, negatively charged.^167^ However, the effect of this charge on the optical properties of Chl C4 is not strong,^131^ while a similar analysis for Chl B7 is still pending. Next to the Glu, there is a Tyr in CP47 forming a hydrogen bond to the 13^1^‐keto group of Chl B7, whereas there is a Phe in CP43 that is not able to donate such a bond to Chl C4. However, in both binding sites, there are galactolipids. Lipids are integral parts of PSIIcc as reviewed elsewhere.^56^, ^57^, ^58^ The location of the galactose head groups of these lipids remained somewhat obscure at a resolution of 3 Å. The new cyanobacterial structure shows that a monogalactosyldiacylglycerol (MGDG) lipid competes with Tyr B40 for hydrogen bonding with Chl B7. Two lipids, a MGDG and a digalactosyldiacylglycerol, are close to Chl C4, but only the MGDG forms a hydrogen bond. Such pigment–lipid interactions can have a profound influence on the optical properties of the Chls as discussed earlier, where it was even proposed that lipids could play a role in causing red shifts of Chl absorptions.^167^ A problem is that these interactions are perturbed, when PPCs are solubilized in detergent solution. In particular, preparation of subcomplexes such as CP43 or CP47 from plants may result in lipid extraction and entail alterations of the optical spectra that are difficult to assess.

A peculiar type of pigment–protein interaction is found for Chl C4, where Trp C63 forms an NH‐π bond with the tetrapyrrole ring system. Electrostatic computations predict this special hydrogen bond to cause a blue‐shift of the corresponding Chl absorption,^131^, ^167^ but it remains to be clarified, whether the electrostatic model is sufficient to describe such an interaction. Another special property of Chl C4 is that is has been suggested to possess a farnesyl tail (15 carbon atoms) at the 17^3^‐ester instead of the common phytyl chain (20 carbon atoms) based on a refinement of the 3ARC/3WU2 structure at a resolution of 1.9 Å (refined to 5V2C, see Table 1).^45^ In the RT structure 6DHE, the Chl C4 chain is modeled with 14 carbon atoms and rather as a truncated phytyl than a farnesyl tail (Figure 7b). A problem is the presence of lipids in the binding site of Chl C4, making it difficult to disentangle the phytyl/farnesyl and the fatty acid alkyl chains.

The diatom structure features three new Chl pigments in the periphery of the core complex probably connecting the core antenna to the outer antenna system. Chl G1 is bound to PsbG (Figure 6a), which could so far be modeled only as poly‐alanine, so that the axial ligand and other possible interactions of Chl G1 with PsbG remain obscure. As regards the layer structure of Chls, Chl G1 belongs to the stromal layer (Figure 6a), but is relatively remote from the Chls of CP47. Instead, it is located close to the Chls of the ST antenna complex (Figure 3b). Chl W1 also belongs to the stromal layer, but in contrast to G1, it can be considered as part of the core antenna system as it is located close to CP43 pigments (Figure 5b). The axial ligand is a Glu residue from PsbW, while the hydrogen bond donor to the 13^1^‐keto group is Trp W114 (Table 2). Chl Z1 is bound to PsbZ, belongs to the luminal layer and is located close to the FCP‐E antenna (Figure 3b).

## 5 STRUCTURE‐BASED COMPUTATIONS

Linking structure and function of PPCs is complicated by the fact that the singlet excited states formed after photon absorption, also referred to as exciton states, cannot in general be assigned to individual pigments. The reason for this exciton delocalization is that the electrons of two pigments “feel” each other, if the two molecules are not too distant. As a consequence, if one pigment becomes excited, neighboring pigments become also excited, so that one can no longer say, where actually the exciton is. If the PPC is in a given exciton state, all that can be said is that a pigment contributing to this state is excited with a certain probability. The experimental manifestation of these quantum physical effects is that optical bands are shifted and changed in intensity with respect to the bands of the uncoupled pigments. We will see examples below in the context of the RC. Furthermore, the exciton delocalization also affects the way the energy is transported through the antenna system.^51^, ^52^, ^53^ Therefore, in order to understand optical spectra and EET, we need to know two types of parameters: site energies and excitonic couplings. The site energy is the energy difference between the first excited and the ground state of an individual pigment in its binding site in the PPC. If the pigment was not coupled to other pigments, it would give rise to an absorption band at this energy. However, due to the mutual perturbation of the electrons from different pigments, which is quantified by the excitonic coupling, the bands are modified as described above, and information about the individual pigments can no longer be directly inferred from the experimental spectra. This problem would even occur, if the spectra were not congested because of the large number of pigments absorbing in a rather narrow wavelength range.

The structure‐based computation of excitonic couplings is relatively easy compared to site energies. Details can be found in earlier reviews.^13^, ^53^ There are several methods to obtain site energies from structural information that apply QC at various degrees of sophistication and that all have their advantages, disadvantages, and accuracy.^53^, ^54^, ^55^ Frankly speaking, there is actually no method that is really accurate enough, and there is always the need for some readjustment of the site energies to achieve a reasonable agreement between simulated and measured optical spectra. Nonetheless, structure‐based computations help to reduce the ambiguity of the site energy assignment considerably. As mentioned in Section 1, we will focus here on electrostatic methods, specifically on the Poisson–Boltzmann/Quantum‐Chemical (PBQC) method.^28^, ^168^, ^169^, ^170^

**Figure 8:**
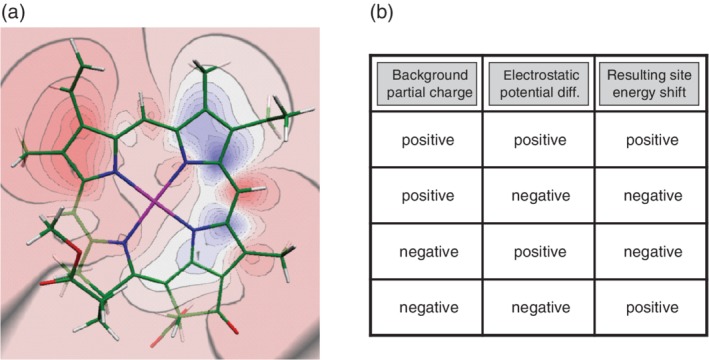
(a) Electrostatic potential difference between the first excited and the ground state of Chl *a* as contour plot in the π‐plane of the molecule (blue, positive; red, negative) obtained from quantum chemical computations (HF‐CIS).^170^ (b) Contribution of a background partial charge to the resulting site energy shift of a pigment depending on the relative sign of the background partial charge and the difference potential

In the PBQC method, one starts with a QC computation of the plain pigment (e.g., Chl *a*) in vacuum with an optimized molecular structure to determine the electrostatic potential of the molecule in its ground (*S*_0_) and in its first excited (*S*_1_) electronic state. The *S*_1_ – *S*_0_ difference of this potential is shown in Figure 8a for Chl *a*. As can be seen, excitation of Chl *a* leads to a significant redistribution of electrons within the molecule (from the blue to the red regions). This redistribution gives rise to an electrostatic potential difference that interacts with the protein environment, thus causing a site‐specific shift of the energy difference between *S*_1_ and *S*_0_ state, that is, the site energy shift. To facilitate the computation of this shift, an atomic partial charge (APC) is assigned to each atom of Chl *a* in a way that the QC‐derived electrostatic potentials are reproduced. These APCs are then used as source terms in the numerical solution of the Poisson–Boltzmann equation. This equation determines the electrostatic potential that a certain charge distribution produces within a polarizable medium including mobile ions. In the actual computation, one assigns different dielectric constants to the protein volume, the membrane interior, and the surrounding aqueous medium.^53^, ^170^ In addition, the latter is characterized by an ionic strength representing the mobile charges. The electrostatic potential resulting from the solution of the Poisson–Boltzmann equation is finally used to compute two contributions to the site energy shift. One is the interaction of the original charge distribution of the pigment with the dielectric polarization it induces in the protein environment, known as the reaction potential. The second is the interaction with background charges, that is, APCs that are located on protein atoms and usually are derived from parameter sets used in molecular dynamics (MD) simulations. This latter contribution to the site energy shift is the most important one. It can be memorized by the simple scheme depicted in Figure 8b. According to this scheme, for example, a positive background charge (e.g., from the hydrogen atom in a hydroxy group) placed in the negative region of the difference potential (e.g., close to the 13^1^‐keto group of Chl *a*) causes a negative site energy shift, that is, a red‐shift of the corresponding absorption band (e.g., due to a hydrogen bond to the 13^1^‐keto group).

The PBQC method has clear advantages, but also limits. One advantage is that only one QC computation, which is usually time‐consuming, has to be made for a certain type of pigment, whereas all site‐specific properties are computed by electrostatics, which is much faster and can be done for all sites in one run. As a result, computations of systems as large as a core complex or probably even a supercomplex, which contain many pigment sites, become feasible in a reasonable time. This simplification comes at a price, however: The site‐specific influence of the protein on the electronic structure of the pigments (and hence on the APCs assigned to the pigment states) is neglected. Conformational variations of the pigments are taken into account only insofar as the APCs are placed on atom positions derived from the experimental structures (except for hydrogen atoms, where the positions are modeled).

Another advantage of the PBQC method is that the APCs assigned to the environment of a pigment can be “switched off” in order to identify parts of the PPC that are relevant to determine a certain site energy shift. In this way, a number of amino acid side residues in CP43 have been proposed as targets for mutagenesis experiments that might help to evaluate the site energy assignment.^131^, ^167^ The problem remains that specific pigment‐protein interactions might not be adequately described by electrostatics such as the interaction of Trp C63 with Chl C4 (see above), which requires further investigations. Another disadvantage of the PBQC method is that it is static. While being faster than a site‐by‐site QC computation, the numerical solution of the Poisson–Boltzmann equation is still relatively slow so that it cannot easily be combined with MD. Thus, the PBQC method relies on the static crystal or EM structure. The result depends on the quality of the structure (which is why we are not interested in structures with a resolution less than about 3 Å) and on the extent to which the structure represents the average of structures present in the samples used to record optical spectra. The latter problem is of particular relevance in studies of subcomplexes of dPSIIcc, where disintegration of the core complex might cause structural alterations of pigment sites. However, problems of this kind also occur with seemingly intact complexes. In the case of LHCII, evidence was found that a pigment might have a different orientation in the crystal structure than in typical samples used for optical spectroscopy.^171^ Jankowiak and coworkers collected plenty of data indicating sample heterogeneity and a possible lack of intactness.^142^, ^159^

As regards the PSII core complex, the PBQC method has been applied so far to isolated CP43, CP47, and the RC. The results are compiled in the column “PBQC” of Table 2. The RC is further discussed below. In the case of CP43, a satisfactory agreement between simulated and measured optical spectra of various types (i.e., absorption, fluorescence, linear dichroism (LD), circular dichroism (CD), circularly polarized luminescence (CPL)) could be achieved after adding to the PBQC results obtained with cyanobacterial structures a slight readjustment of site energies to fit the experimental data of cyanobacterial and plant material.^131^, ^172^ The situation is less pleasant in the case of CP47, where the PBQC computations are in an intermediate stage, and the site energy assignment made^132^ is more controversial.^142^ The discussion of this problem is continued below in the context of “red chlorophylls.”

The PBQC method has a “younger brother” introduced by Adolphs et al.^169^ and termed the Charge Density Coupling (CDC) method. In this method, only pairwise Coulomb interactions between APCs of the pigments and the protein environment are computed taking into account an effective dielectric constant.^53^ This method is much faster than the tedious numerical solution of the Poisson–Boltzmann equation and was shown to give comparable results when applied to static structures.^131^, ^173^ Accordingly, the CDC method was combined with MD simulations of PPCs to obtain site energy shifts for a large number of structural variants that occur at physiological temperatures and also to study the coupling of pigment excited states to vibrations of the PPC.^174^ Recently, this method was applied to dPSIIcc from *Th. vulcanus*,^133^ and the resulting site energies are compiled in the column “CDC” of Table 2. It should be noted that these site energy values were not yet tested against low‐temperature optical spectra of various types.

As regards the structure‐based simulation of optical spectra using electrostatic methods, current research focuses on the improvement of the QC‐description of chlorophylls, simulations of the spectral density of exciton‐vibrational coupling,^175^ and simulation methods to sample slow conformational changes that give rise to inhomogeneous broadening of optical bands due to static disorder.

## 6 THE REACTION CENTER

Application of the PBQC method to the RC of PSII^28^ confirmed an earlier assignment of site energies,^136^, ^176^, ^177^ in which Chl_D1_ has the lowest site energy in the RC, that is, it is lower by ~100 cm^−1^ than that of the symmetry‐related Chl_D2_, and there is a kind of reversed asymmetry at the pheophytin level, that is, the site energy of Pheo_D1_ is higher than that of Pheo_D2_ (Figure 1, Table 2). Qualitatively, the same result is obtained with the CDC/MD method^133^ (Table 2). It should be emphasized that the PBQC‐derived site energies are approved by comparison with a vast amount of experimental spectroscopic data, including besides absorption, fluorescence, and LD also data of site‐specific mutations.^136^, ^137^, ^176^, ^177^, ^178^ (Understanding CD spectra of the RC is still problematic^28^ and may require the inclusion of higher excited states of the pigments.^179^) Although the Chl site energies of the RC appear to be widely accepted now, the Pheo site energies have been more controversial.^180^ In our view, the electrochromic effect of Q_A_ reduction is clearly in favor of a high site energy of Pheo_D1_,^28^, ^181^ but it is certainly worthwhile to further investigate this issue, for example, by simulations of mutants.^28^, ^180^ Another problem is that Chlz_D1_ and Chlz_D2_ are likely lost or spectrally shifted in RC preparations,^182^ making it difficult to benchmark their site energies against experimental data. Note that these two Chls should not be considered as part of the actual RC, although they are harbored by PsbA and PsbD, respectively. In simulating the optical spectra of the RC, one encounters the further problem that the excitonic couplings computed by electrostatic methods^13^, ^51^, ^53^ are insufficient for the P_D1_‐P_D2_ pair, as these two pigments interact closely enough to allow for some electron exchange between them.^183^, ^184^ As a consequence, the excitonic coupling is increased and the exciton states are likely coupled to charge‐transfer (CT) states.^185^

**Figure 9:**
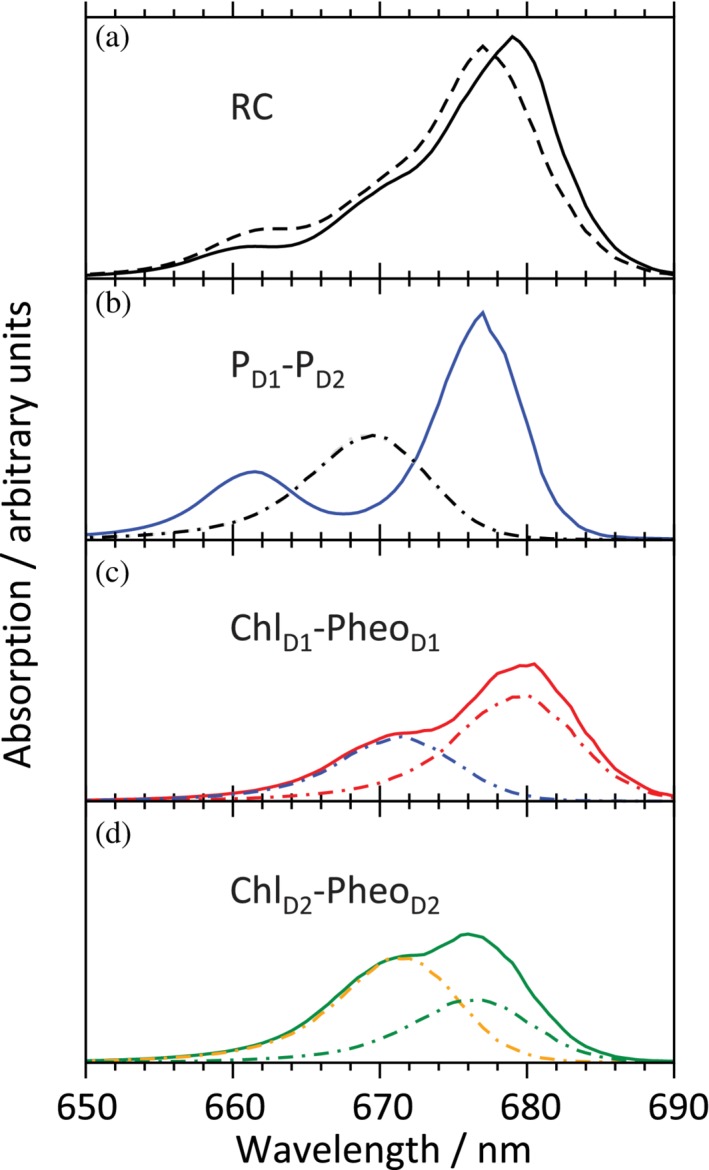
Simulated absorption spectra of the RC in PSII without Chlz_D1_/Chlz_D2_ (a) as well as hypothetical dimers P_D1_‐P_D2_ (b), Chl_D1_‐Pheo_D1_ (c), and Chl_D2_‐Pheo_D2_ (d) with the same excitonic couplings and site energies as in the simulation of the complete RC.^28^ The dashed spectrum in (a) is the sum of the solid spectra in (b)–(d). The dashed‐dotted spectra in (b)–(d) are the spectra of uncoupled pigments with the same site energies. Note that the oscillator strength of Pheo *a* is smaller than that of Chl *a*

Figure 9 gives an impression of how the optical absorption spectrum of the actual RC (i.e., without Chlz_D1_ and Chlz_D2_) should look like (solid spectrum in Figure 9a) according to the site energies given in Figure 1. In these simulations, a value of 158 cm^−1^ was assumed for the excitonic coupling between P_D1_ and P_D2_ (larger than the electrostatically computed value) and no coupling to CT states was considered.^28^ In accordance with experimental data, the absorption spectrum has a maximum near 680 nm. This band is bleached, when light‐triggered CS takes place in the RC. Accordingly, the term “P680” was coined, referring to a pigment (P) with an absorption maximum at 680 nm. By analogy with P700 of PSI and P865 of PBRC, “P680” is interpreted as the primary donor of the CS process that is oxidized to “P680^+^.” Accordingly, it is the “P680/P680^+^” redox couple that is considered to be the species with the high midpoint potential of more than 1 V that is suitable for water oxidation. This line of interpretation, based on the analogy to P700 and P865, also implies that the P_D1_‐P_D2_ pair is the analogue of the “special pair” of PBRC.^183^ Already a long time ago, it has been discussed that such an interpretation of the term “P680” is untenable.^186^ Nonetheless, it is often found in the literature even nowadays. However, the RC of PSII is different.

First, the absorption band at 680 nm has contributions from all six pigments in the RC. If these six pigments were not interacting by excitonic couplings, they would show the absorption spectra drawn with dash‐dotted lines in Figure 9. Let us now have a closer look at P_D1_ and P_D2_. Both pigments have the same site energy (at least according to our simulations). The strong excitonic coupling between P_D1_ and P_D2_ (which likely has an electron‐exchange contribution) causes a splitting of the absorption into two bands of different intensity (solid line in Figure 9b). This splitting of bands due to the presence of more than one equivalent molecular entity is referred to as Davydov splitting and is a typical quantum effect. If, for example, the band at 677 nm is excited, one cannot say, whether P_D1_ or P_D2_ is excited. In the case of the other two simulated dimers, that is, Chl_D1_‐Pheo_D1_ (Figure 9c) and Chl_D2_‐Pheo_D2_ (Figure 9d), the splitting effects are not so clearly visible, but there are still significant intensity changes. Note that adding up all three “dimer spectra” does not result in the proper spectrum of the RC (Figure 9a), because there are additional excitonic couplings between the pigments that ultimately give rise to the absorption maximum near 680 nm. So, whatever “P680” is, it is not a single pigment, but rather the whole RC.

Because of its relatively strong coupling, the P_D1_‐P_D2_ pair is indeed somewhat “special,” but the coupling is significantly weaker than in its counterparts in PSI and PBRC.^183^, ^184^ There is evidence from simulations of difference spectra, that after CS the hole is localized on P_D1_ (leaving the possibility of a slight delocalization of the hole onto P_D2_).^176^ Thus, if one accepts that a pigment pair with an absorption band at 677 nm (like P_D1_‐P_D2_) is labeled “P680,” then one could still speak of a special pair in PSII that is the strong oxidant required for water oxidation. However, this special pair is neither the primary electron donor (or at least not exclusively, see below), nor does it give rise to a bleaching at 680 nm during the CS process. Evidence for the latter originates from spectroscopic studies of site‐directed mutations of PSII from *Synechocystis* sp. PCC 6803.^178^ If the axial ligand of P_D1_, His A198 (Table 2), is replaced with Gln, the difference spectra are changed, but the bleaching at 680 nm is not. Accordingly, if Thr A179, which is in contact with the water molecule axially ligating Chl_D1_, is changed to His, the bleaching band at 680 nm is shifted. This result suggests that the mutation at position A179 mainly affects Chl_D1_, which has a strong contribution to the absorption at 680 nm (Figure 9). These mutations also helped to identify Chl_D1_ as the location of the triplet state of the RC. The triplet‐minus‐singlet difference spectrum has a negative peak slightly above 680 nm, giving rise to the impression that a triplet state of a pigment “P680” is formed. Thus, we have the curious situation that the triplet state of “P680” is actually the triplet state of Chl_D1_, while the oxidized state of “P680” is actually P_D1_^+^ and the excited singlet state of “P680” is actually a linear combination of excited states of all six pigments in the RC. We come to the conclusion that the term “P680” is not very useful (in contrast to its meaningful counterparts P700 and P865) and should be put aside.

What about the primary electron donor? Given the site energies and excitonic couplings, one can compute the probability to find a pigment excited at a certain temperature. Based on the site energy assignment of the RC confirmed by the PBQC method, these probabilities have been computed for temperatures of 5 and 300 K.^137^, ^177^ At 5 K, almost 90% of the excitation energy is located on Chl_D1_ suggesting that the excited state of this pigment is the precursor of CS and, hence, that Chl_D1_ is indeed the primary electron donor. The first step of CS would then be ET from Chl_D1_ to Pheo_D1_. Subsequent steps would be hole transfer from Chl_D1_ to P_D1_/P_D2_, ET from Pheo_D1_ to Q_A_ and, finally, ET from Q_A_ to Q_B_. Note that Q_B_ is the final electron acceptor that after taking up two electrons and two protons leaves the RC into the thylakoid membrane. In this scenario, the asymmetry of the excited states would lead to the asymmetry in ET that is required to specifically reduce one of the PQs, and the “special pair" (P_D1_/P_D2_) would still be the strong oxidant for water splitting. Unfortunately, the situation is not as straightforward under more physiological temperature conditions. At 300 K, the excitation energy is more evenly distributed over the RC pigments^137^, ^177^: 30% on Chl_D1_ (still prevailing), 20% in Pheo_D2_, 15% on P_D1_ and on Pheo_D1_, approximately 10% on P_D2_ and on Chl_D2_. These numbers do not allow for conclusions about the CS mechanism under real‐life conditions. In fact, it has been proposed that besides the sequence described above, an alternative CS pathway starting at P_D1_/P_D2_ is operative (see Novoderezhkin et al.^187^ and references therein), but a final proof is still missing.

We have not yet discussed one particular interesting aspect of the PBQC method: It allows for the determination of protonation states of titratable groups in the protein. Indeed, the PBQC method originates from earlier computational schemes for protonation states.^53^, ^188^, ^189^ The application of the PBQC method to the RC of PSII was based on a structural model of the D1‐D2‐cyt*b*_559_‐PsbI‐complex.^28^ Experimentally, it is known that water oxidation is impaired in such RC preparations, which is likely due to a loss of the metal ions of the WOC. To investigate possible consequences of such a loss, two types of simulations were conducted: either with an intact WOC or with the metal ions removed. The Mn_4_CaO_5_ cluster is coordinated by seven amino acids: Asp A170, Glu A189, His A332, Glu A333, Asp A342, Ala A344, and Glu C354.^14^, ^19^, ^26^, ^44^ Six of these metal ligands are negatively charged carboxyl groups (side chains except for Ala A344, where the ligand is the C‐terminus of PsbA). Since the intact Mn_4_CaO_5_ cluster is well charge‐balanced, removal of the metal ions would result in the accumulation of up to six negative elementary charges in the WOC site. Possible consequences are (a) a destabilization of the protein and (b) significant electrochromic shifts of Chl site energies in the RC. Indeed, model calculations demonstrate that redshifts of site energies would result, for example, of Chl_D1_ by approximately 80 cm^−1^.^190^ Given that the asymmetry of site energies in the RC is rather subtle (Figure 1, Table 2), and the energy shifts of charge‐separated states can be expected to be an order of magnitude larger than those of excited states, such perturbations could sensitively disturb RC function. However, there is evidence that this problem does not occur: (a) crystallographic studies of *apo*‐PSII (i.e., dPSIIcc with the metal ions removed after crystallization) suggest that the WOC binding site remains structurally intact even without metal ions (containing water molecules instead).^44^ This finding argues against a destabilization of the protein. (b) Simulations of optical spectra comparing PSIIcc (with intact WOC) and RC (with the metal ions likely removed) suggest that the site energy of Chl_D1_ is rather blue‐ than red‐shifted in the RC.^138^, ^177^ Whatever the reason for this shift is, it can hardly be explained by the electrochromic effect of negative charges in the WOC site (and it also does not impair RC function). So, what is going on? Solving this riddle is quite simple: The PBQC‐computations suggest that the carboxyl groups of the metal ligands become largely protonated after metal removal, which results in charge compensation.^28^, ^190^ Zhang et al.^44^ arrive at a similar conclusion in their analysis of the *apo*‐PSII structure.

What about the accumulation of positive charges? The catalytic cycle of the WOC involves removal of an electron from the Mn_4_CaO_5_ cluster in each step, and the electron is transferred via the redox‐active tyrosine Y_Z_ to P_D1_^+^ (cf. Figure 1). Hence, up to four positive elementary charges could be accumulated in the WOC site and impair the RC. Again, this problem does not occur: The investigation of electrochromic shifts showed that maximally one positive surplus charge is accumulated in the WOC site during the water oxidation cycle.^191^, ^192^ The explanation is the same as above: changes of protonation states. Amino acid side chains in the vicinity of the Mn_4_CaO_5_ cluster release protons into the lumen.^14^, ^193^, ^194^ Electrostatic computations show that the excited states of the RC are only marginally affected by one positive surplus charge in the WOC site.^190^ These findings are in agreement with the hypothesis stated above that water oxidation does not affect the RC in order to guarantee proper CS irrespective of the redox state of the WOC. In this way, the four‐electron chemistry in the Mn_4_CaO_5_ cluster can be linked to the one‐electron processes in the RC, which in turn are connected to the two‐electron reduction of Q_B_.^26^

If the RC works properly even in the absence of an active WOC, a new problem arises: Charge‐separated states involving P_D1_^+^ may accumulate and damage PSII, a phenomenon known as donor side mediated photodamage.^195^ This problem calls for a protection mechanism. Based on fluorescence experiments, it was found that removal of the Ca^2+^ ion from the Mn_4_CaO_5_ cluster apparently changes the redox midpoint potential of Q_A_.^196^ Accordingly, a mechanism was proposed, in which a shift of the energy level of the P_D1_^+^Q_A_^−^ state opens a channel for a radiationless decay to the ground state in order to decrease the lifetime of potentially harmful radical pair states. Very recently, Fourier‐transform infrared (FTIR) spectroelectrochemistry experiments showed that Ca^2+^‐removal does not affect the redox midpoint potential of Q_A_.^197^ The reason for the discrepancy is that the fluorescence data do not directly reflect the redox state of Q_A_, whereas FTIR yields more direct information. Thus, inactivation of the WOC does not affect the RC in accordance with our hypothesis.

These findings do not imply that there are no shifts in the redox potential of Q_A_. There is evidence that this potential is influenced by other factors such as the redox state of Q_B_ and the quinones in the thylakoid membrane (quinone pool), herbicide binding to the Q_B_ site or bicarbonate binding to the non‐heme iron.^17^, ^30^, ^198^, ^199^ Such a redox tuning of Q_A_ could be involved in a phenomenon known a “reaction center quenching,”^200^ that is, a deactivation of excited states of the antenna via charge‐separated states in the RC. From the viewpoint of light harvesting, such an RC‐based regulation mechanism can be considered as photochemical quenching, which is different from the non‐photochemical quenching^85^, ^88^, ^89^ occurring in the peripheral antenna.

## 7 ASSIGNMENT OF “RED CHLOROPHYLLS”

Based on the above analysis, the “red Chls” that we are concerned with here are located in CP47, should be in contact with carotenoids, and at least some of them should interact with PsbH. Before proceeding, we have to consider another factor influencing optical spectra: molecular vibrations. Whereas the excitonic couplings tend to delocalize excitons (depending on site energy differences), the molecular vibrations perturb the electrons of the pigments in a way that the exciton delocalization becomes time‐dependent and the exciton states involving weakly coupled pigments become more localized. A theoretical modeling of such dynamical localization effects is demanding and presently not feasible for large PPCs. Therefore, an approximation is used, in which time‐independent exciton delocalization is assumed, but the delocalization is allowed only within groups of strongly coupled pigments. These groups are referred to as domains (or exciton domains) and are defined by a threshold value *V*_c_ for the magnitude of the excitonic coupling. Thus, pigments belonging to different domains have an excitonic coupling smaller in magnitude than *V*_c_. Note that all changes of peak positions and intensities discussed above (for the example of the RC) only occur within one exciton domain, whereas the optical spectra of the domains simply add up to give the total optical spectrum of the PPC.

In recent simulations of CP47,^132^, ^138^, ^142^ five exciton domains are distinguished. According to Hall et al.,^132^ the domains containing more than one pigment are labeled R (containing Chls B1 to B3, B5, and B6), G (B4 and B7) as well as C (B8 to B14). In addition, Chls B15 and B16 form domains on their own, since all excitonic couplings of these two pigments are smaller in magnitude than *V*_c_ = 30 cm^−1^. Note that within this approximation, the latter two pigments do not show up in CD and CPL spectra as the signals detected in this type of spectroscopy require excitonically coupled pigments.^201^ This fact was exploited by Hall et al.^132^ in their spectroscopic analysis of isolated CP47 from plants. CPL showed that red‐shifted states involve excitonically coupled Chls. Together with LD spectra, this information was used to rule out B16 as “red Chl” in favor of B1. This assignment was challenged by Reinot et al.,^142^ who offered several alternative site energy assignments including different red‐shifted Chls such as B11, B13, and B16. Given that the plant preparations very likely do not contain PsbH and are structurally perturbed, it is difficult to draw any conclusions about red‐shifted Chls from all these data.

In a different set of experiments, Skandary et al.^143^ investigated dPSIIcc and mPSIIcc from *Th. elongatus* by using single‐molecule spectroscopy (SMS). They found a significant reduction of red emitters in mPSIIcc compared to dPSIIcc. Since the crystal structure of mPSIIcc showed that Car t1 is missing,^67^ it was argued that Chl B7 could be a red‐shifted Chl.^143^ The rationale is as follows: If a carotenoid (t1) strongly interacting with the Chl (B7, cf. Table 3) is lost, it can no longer quench the triplet state of that Chl, which opens a channel to depopulate the singlet state of the Chl under the conditions of SMS (which is different from conventional fluorescence spectroscopy) and, hence, the singlet emission is reduced. Problems with this interpretation are that (a) Car t1 might also be missing in some dPSIIcc preparations, and (b) carotenoids at the monomer–monomer interface other than Car t1 might be lost in mPSIIcc. These uncertainties allow for alternative assignments of the red emitters, for example, Chl B16.

Based on the criterion of strict van der Waals contact with a carotenoid at physiological temperatures, Chls B9 and B14 should be additionally considered as long‐wavelength Chls (see above). Given that a hydrogen bond to the 13^1^‐keto group causes a red‐shift, the data of Hall et al.^132^ and D'Haene et al.^157^ could be reconciled by assuming that a loss of PsbH in the plant preparations causes a blue‐shift of the site energies of B9 and B16 due to removal of the hydrogen bonds, while B1 is red‐shifted anyway and remains unaffected by the loss of PsbH. This model predicts the presence of three different red‐shifted emitters in intact core complexes. Only one of these emitters corresponds to the localized excited state of a single Chl (B16). The other two emitters are actually exciton states of a small (R) and a large (C) exciton domain, which should give rise to detectable features in CPL spectra that partly depend on the subunit composition of the sample.

As stated above, structure‐based site energy computations of CP47, although already used in simulations,^132^ still need further refinement and, in the future, will also have to include structural information about species other than cyanobacteria, which is now available (Table 1). In any case, a lot of work remains to be done to clarify the identity of the “red Chls,” not to mention their functional role.

## 8 EXCITATION ENERGY TRANSFER

There has been a long‐lasting debate about the question, how the excitation energy in the core antenna of PSII is trapped in the RC.^13^ In the excited‐state‐radical‐pair‐equilibrium (ERPE) model, it is assumed that equilibration of excited states in the whole core complex (i.e., including exciton states located on the RC, CP43, and CP47) is fast compared to the primary CS occurring in the RC.^202^, ^203^ In this model, energy trapping is kinetically limited by the CS reaction in the RC (trap‐limited). However, studies reporting a relatively slow EET between CP43, CP47, and the RC on the 20–30 ps time scale^204^, ^205^ and a relatively fast primary CS in the 600–800 fs range^206^ challenged the ERPE model. If primary CS is much faster than EET, the whole process is transfer‐to‐the‐trap limited. It has also been suggested that CS and EET occur on the same time scale.^207^ Structure‐based simulations support the transfer‐to‐the‐trap model, as the fast equilibration suggested by the ERPE model is not compatible with the large distance between antenna and RC pigments.^136^, ^138^, ^208^ Thus, the available structural information could have concluded the debate, but apparently it did not. There are two recent experimental studies that provide again support for the transfer‐to‐the‐trap model by demonstrating the relatively slow EET to the RC. In one experiment, oriented single crystals of dPSIIcc from *Th. elongatus* are excited by polarized visible light, and the transient absorption is probed with polarized light in the infrared region.^209^ Analysis of the time‐dependent dichroism in the infrared provided evidence for a 50–100 ps equilibration between CP43 and CP47 across the RC. In the second type of experiment, solubilized PSIIcc was excited around 500 nm to induce EET from carotenoids to Chls.^210^ The data suggest that CS can be induced by direct excitation of Car_D2_ in 9.6 ps, whereas all other carotenoids transfer energy to Chls in CP43 and CP47, from where it is delivered to the RC in 20 ps.

## 9 CONCLUDING REMARKS

During the last 12 years, a remarkable progress has been made in elucidating the structure of PSII and its core complex. However, the major part of this progress is relatively recent, so that the understanding and functional interpretation of the structural features lag behind. This delay in exploiting the structures is particularly evident in the field of light‐harvesting, where the interpretations significantly rely on theoretical simulations. A bottleneck in these simulations is the quantum chemical description of the optical properties of chlorophylls, which in one or the other way is central to all theoretical models of light‐harvesting. Another challenging task is to include molecular dynamics in the simulations, which for systems as large as dPSIIcc is emerging as a viable way to reach insights into the working principles of oxygenic photosynthesis.
